# CASTIN: a system for comprehensive analysis of cancer-stromal interactome

**DOI:** 10.1186/s12864-016-3207-z

**Published:** 2016-11-09

**Authors:** Daisuke Komura, Takayuki Isagawa, Kazuki Kishi, Ryohei Suzuki, Reiko Sato, Mariko Tanaka, Hiroto Katoh, Shogo Yamamoto, Kenji Tatsuno, Masashi Fukayama, Hiroyuki Aburatani, Shumpei Ishikawa

**Affiliations:** 1 Department of Genomic Pathology, Medical Research Institute, Tokyo Medical and Dental University, Tokyo, Japan; 2 Graduate School of Interdisciplinary Information Studies, The University of Tokyo, Tokyo, Japan; 3 Graduate School of Information and Science and Technology, The University of Tokyo, Tokyo, Japan; 4 Department of Pathology, Graduate School of Medicine, The University of Tokyo, Tokyo, Japan; 5 Genome Science Division, Research Center for Advanced Science and Technology, The University of Tokyo, Tokyo, Japan

**Keywords:** Cancer microenvironment, Cancer-stromal interactions, Xenograft mouse model, RNA-Seq

## Abstract

**Background:**

Cancer microenvironment plays a vital role in cancer development and progression, and cancer-stromal interactions have been recognized as important targets for cancer therapy. However, identifying relevant and druggable cancer-stromal interactions is challenging due to the lack of quantitative methods to analyze whole cancer-stromal interactome.

**Results:**

We present CASTIN (CAncer-STromal INteractome analysis), a novel framework for the evaluation of cancer-stromal interactome from RNA-Seq data using cancer xenograft models. For each ligand-receptor interaction which is derived from curated protein-protein interaction database, CASTIN summarizes gene expression profiles of cancer and stroma into three evaluation indices. These indices provide quantitative evaluation and comprehensive visualization of interactome, and thus enable to identify critical cancer-microenvironment interactions, which would be potential drug targets.

We applied CASTIN to the dataset of pancreas ductal adenocarcinoma, and successfully characterized the individual cancer in terms of cancer-stromal relationships, and identified both well-known and less-characterized druggable interactions.

**Conclusions:**

CASTIN provides comprehensive view of cancer-stromal interactome and is useful to identify critical interactions which may serve as potential drug targets in cancer-microenvironment. CASTIN is available at: http://github.com/tmd-gpat/CASTIN.

**Electronic supplementary material:**

The online version of this article (doi:10.1186/s12864-016-3207-z) contains supplementary material, which is available to authorized users.

## Background

Cancer cells generally survive in microenvironment surrounded by non-cancer “stromal” cells such as endothelial cells, fibroblasts and immune cells. Stromal cells in cancer microenvironment promote maintenance, growth and progression of cancer cells through the release of humoral factors and direct cell contact. Conversely, cancer cells promote fibroblast proliferations, immune cell migration and angiogenesis through signal transduction. Thus cancer microenvironment is regarded as a key contributor for epithelial-mesenchymal transition of the cancer cells, angiogenesis, cancer progression and metastasis, and development of drug resistance [1].

Recently, there has been a growing interest in targeting cancer microenvironment for cancer treatment [1–4]. Inhibition of cancer stromal interaction may prevent neovascularization, invasion, and metastasis and improve anti-cancer drug delivery. For example, inhibition of Hedgehog signaling improves delivery and efficacy of gemcitabine in a mouse pancreatic cancer model [5]. However, compared to targeting driver ‘mutations’ which are tractable by genome-wide comparison of mutation frequency [6], exploration of driver ‘interactions’ is far more challenging due to the exponential number of possible interactions between proteins and lack of high-throughput methods that can quantitatively interpret the cancer-stromal interactions.

Xenograft cancers from human-derived cells grown in immune-compromised mice have been extensively used to study cancer and its microenvironment [7–12]. Xenograft cancers establish microenvironment by inducing mouse-derived stromal cells such as fibroblast and vascular cells, and can closely resemble the original cancer growing in a patient [8]. Given that there is approximately 15 % sequence difference between human and mouse exon sequences [9], simultaneous transcriptome analysis of cancer and stroma can be achieved using RNA-Seq or species-specific microarray.

Several computational methods have been developed to analyze microarrays or RNA-Seq data from cancer xenograft mouse models. Like conventional single-species gene expression analysis, all of these methods compare gene expression profiles in two conditions, e.g. xenograft vs in cell line, or before vs after the addition of a molecule which would change the cancer-microenvironment [7, 13–15], and subsequently apply Gene Set Enrichment Analysis (GSEA) [7] or pathway analysis [13–15]. These approaches are effective in identifying gene sets or pathways contributing to the change. However, they have several limitations. First, since expression profiles of cancer cells and stromal cells are treated independently, interactions between them cannot be explicitly evaluated. Second, GSEA and pathway analysis only provide induced change as a whole, thus individual interactions cannot be evaluated. These limitations have created a bottleneck especially when the purpose is to evaluate individual interactions and to prioritize cancer-stromal interactions as the targets for cancer treatment.

To overcome such limitations, we have introduced a novel interactome analysis framework, CASTIN (CAncer-STromal INteractome analysis) for quantitative profiling of cancer-stromal interactome from RNA-Seq data using cancer xenograft mouse models. CASTIN determines direction and strength of individual transmitting signals between two interacting cells based on the expression levels of cancer and stroma. CASTIN focuses on ligand-receptor interactions because they are central to the cellular communication and, more importantly, since they involve cell surface and extracellular molecules, they are accessible by biomolecular drugs such as antibodies, peptides, and aptamers. The ligand-receptor relationships are extracted from public protein-protein interaction databases and they are manually curated. Summarization of each interaction into only three interactome evaluation indices enables us not only to quantitatively compare different interactions and to prioritize one particular interaction for clinical approach, but also to visually interpret the global cancer-stromal interactome of individual cancer and the relative importance of each interaction. To our knowledge, CASTIN is the first computational method to quantitatively evaluate cancer-stromal interactions from RNA-Seq data of cancer xenograft mouse models.

We have demonstrated that CASTIN can successfully characterize the individual cancer in pancreatic cancer in terms of cancer-stromal relationships, and identify both well-known and less characterized important interactions.

## Results and discussion

### A system for cancer-stromal interactome analysis

**Fig. 1:**
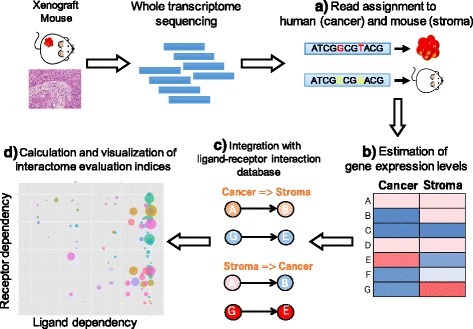
workflow of the CASTIN algorithm. Total RNA extracted from cancer xenograft mouse model, which contains both human and mouse RNA, is sequenced using next generation sequencing. **a** Human (cancer) cell- and mouse (stromal) cell-derived transcripts are differentially assigned. **b** Accurate gene expression level is estimated using GC contents, distance from poly-A tails and originally defined mappable regions among annotated transcripts. **c** gene expression levels are integrated with an in-house ligand-receptor interaction database. **d** three indices (signal strength, ligand dependency, and receptor dependency) for quantitative evaluation of the interaction are calculated for the two directions of signal transduction and plot for visualization

Figure 1 shows the overview of the CASTIN algorithm, which consists of four main steps: (i) read assignment to human (cancer) and mouse (stroma) derived transcripts, (ii) quantification of gene expression level, (iii) integrating the gene expression with our in-house ligand-receptor database, and (iv) calculation and visualization of interactome evaluation indices for individual interactions.

In step (i), reads from cancer xenograft mouse models were assigned to RefSeq transcript sequences of human or mouse. Reads mapped to both species or to multiple genes in either species with the same number of mismatches were excluded from the subsequent analysis.

In step (ii), expression levels of each RefSeq gene in human and mouse were estimated. Although this estimation process resembles standard Transcripts Per Kilobase Million (TPM) approach [16], our strategy differs in the following two aspects:

1. CASTIN normalizes gene expression by mappable length whereas standard TPM normalizes it by transcript length. It is done by using precomputed uniquely mappable regions among human and mouse RefSeq transcript sequences. Since homologous regions are found within species or between human and mouse, some reads are not mapped uniquely and estimating expression levels without considering this may lead to inaccurate results.
2. CASTIN removes read count biases arising from regional GC content and distance from poly-A tail (Additional file 1: Figure S1), whereas standard TPM does not consider them. Although the GC bias was very slight, it has been reported in many literatures that extreme GC content leads to an uneven coverage of the transcripts in the next-generation sequencing [17, 18]. The bias due to the distance from poly-A tail was strong in many samples. This bias is reasonable since the library construction process starts with the generation of poly-A primed cDNAs, and more fragmented the RNA is, the lower the mapped count will be in the regions farther away from the poly-A sites. The extent of RNA fragmentation differs sample by sample, but engrafted cancer tissues or cell lines would be severely influenced by this bias as they frequently show focal necrosis due to ischemia or inflammation.

CASTIN estimates and removes the above biases using a statistical model. Although bias correction was performed in human and mouse simultaneously, library size normalization was performed separately because cancer-to-stromal ratio in each tissue sample was different.

Then in step (iii), gene expressions were integrated to our in-house ligand-receptor interaction database. Note that ligands here included not only humoral factors but also cell surface proteins. We have created a database based on multiple protein-protein interaction databases [19, 20]. As the establishment of high quality ligand-receptor interaction database is critical in CASTIN, researchers in the field of biology curated each interaction by carefully reviewing the original literature describing the validation experiments. Additional file 2: Table S1 lists the 628 interactions used in the current version of CASTIN.

**Fig. 2:**
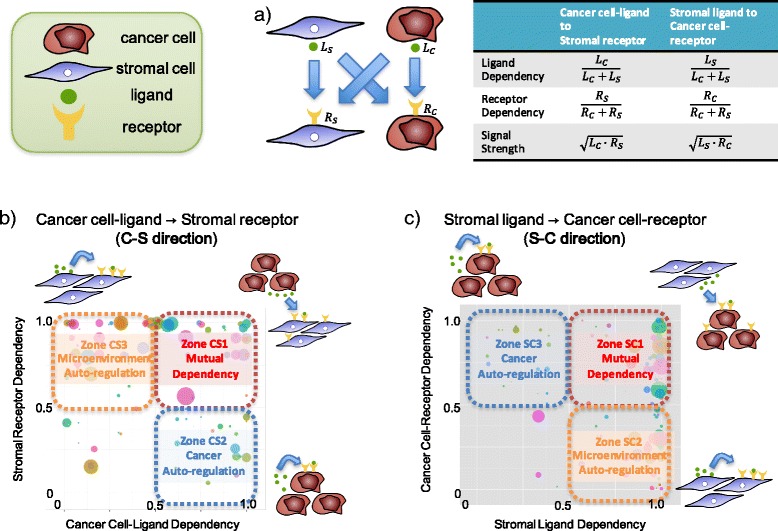
Interactome evaluation indices. **a** Three indices for quantitative evaluation of each interaction. Each index is calculated for two signal directions (C-S direction: cancer ligand to stromal receptor, and S-C direction: stromal ligand to cancer receptor). **b** Visualization of the evaluation indices in C-S direction. X-axis represents cancer cell-ligand dependency and y-axis represents stromal receptor dependency, and positions of interaction indicate the role of the interaction in cancer-stromal relationships, and the size of circle (proportional to log of signal strength) indicates the strength of interaction. **c** Visualization of the evaluation indices in S-C direction, which is the counterpart of C-S direction

In step (iv), three interactome evaluation indices, namely ligand dependency, receptor dependency, and signal strength, were calculated for each interaction (Fig. 2a). The evaluation indices were calculated for the two directions of signal transduction, from cancer ligand to stromal receptor and from stromal ligand to cancer receptor (hereafter referred to as C-S direction and S-C direction, respectively). Ligand dependency in C-S direction, defined as the expression levels of human (cancer) ligand relative to those of human (cancer) plus mouse (stroma) ligand, reflects the dependency of input signal from cancer ligand. Receptor dependency, defined as the expression levels of mouse (stroma) receptor relative to human (cancer) plus mouse (stroma) receptors, is the counterpart of the ligand dependency and reflects the dependency of receiving signal by stromal receptor. Although these two indices are mathematically simple, combination of the two enables us to determine the major direction of signal transduction (from cancer to stroma/cancer, or from stroma to cancer/stroma). Interactions falling into the following six zones in two-dimensional view of ligand and receptor dependency are especially relevant:

1. C-S direction (Fig. 2b):
  Zone CS1 (strong cancer cell-ligand dependency (≥0.5), strong stromal receptor dependency (≥0.5)) : Interactions in this zone indicate that input signal is dominantly created by cancer and exclusively transmitted to stroma. The signal transduction takes place only when both cancer and stromal cells exist, and thus we call it “mutually dependent interaction”.
  Zone CS2 (strong cancer cell-ligand dependency (≥0.5), weak stromal receptor dependency (<0.5)) : Input signal is created by cancer and transmitted mainly to cancer itself. Thus interactions in this zone indicate cancer autoregulation.
  Zone CS3 (weak cancer cell-ligand dependency (<0.5), strong stromal receptor dependency (≥0.5)) : Counterpart of zone CS2. Interactions in this zone indicate microenvironment autoregulation.
2. S-C direction (Fig. 2c):
  Zone SC1 (strong stromal ligand dependency (≥0.5), strong cancer cell-receptor dependency (≥0.5)) : Interactions in this zone indicate mutually dependent interactions. It is similar to zone CS1, but the direction of signal transduction is opposite.
  Zone SC2 (strong stromal ligand dependency (≥0.5), weak cancer cell-receptor dependency (<0.5)) : The direction of the signals is the same as zone CS3, but signal strength is different (See below); interactions in this zone indicate microenvironment autoregulation.
  Zone SC3 (weak stromal ligand dependency (<0.5), strong cancer cell-receptor dependency (≥0.5)) : The direction of the signals is the same as zone CS2, but signal strength is different (See below); interactions in this zone indicate cancer autoregulation.

The remaining regions were designated as Zone CS4 in C-S direction (cancer cell-ligand dependency and stromal receptor dependency are both <0.5) and Zone SC4 in S-C direction (stromal ligand dependency and cancer cell-receptor dependency are both <0.5) for convenience.

In terms of cancer-stromal interactions, probably the most important zones among the 6 zones are CS1 and SC1, where the signals involve specific interaction between receptors of one cell type (i.e., cancer or stroma) and ligands from the other cell type. These “mutually dependent” or “exclusively trans-cell type” signals are potentially important therapeutic targets.

The last index, signal strength, is expressed as the geometric mean of the expression levels of cancer ligand and stromal receptor in C-S direction, or that of stromal ligand and cancer receptor in S-C direction. It is useful to remove weak and possibly non-significant interactions. As the gene expression levels were normalized so that the total expression levels roughly represent the number of mRNA molecules an average cell contains, the signal strength can be used to approximate the average number of mRNA molecules of the interacting genes in two cells.

Using these three indices, one can easily know the signal direction and strength of each interaction, and determine which interactions are critical, that is to say potentially druggable, in cancer-microenvironment relationships. Additionally, overall distributions of three indices, namely the interactome profile, can reflect the sample’s characteristics such as stromal contribution to cancer.

As shown in Fig. 2, these indices are suitable for visualization. To help users customize visualization of the interactome, we have also developed an interactive viewer of CASTIN (http://gpatgazeza.tmd.ac.jp/CASTIN_viewer/, Additional file 3: Figure S2).

### Evaluation of quantification of gene expression level in CASTIN

Unlike the conventional RNA-Seq that deals with single species, RNA-Seq of xenograft involves the separation of human/mouse reads, and we first evaluated this effect on quantification. Using uniquely mappable sequences between human and mouse reference transcripts, human and mouse-derived reads can be differentially assigned to each species. However, sequencing errors, mapping errors and nucleotide sequence variation could lead to misassignment. To investigate the rate of misassignment, cell lines derived from human cancer cells and mouse endothelial cells, both of which do not contain transcripts from any other species, were applied to CASTIN (Table 1). The misassignment rate was 0.0053–0.0124 % in human cell lines and 0.0056–0.0330 % in mouse cell lines (Table 2). Thus the effect of misassignment is considered to be negligible unless content of one of the species is extremely small, where interactome analysis is essentially unsuitable. In the CASTIN system, we have employed a deterministic approach when assigning sequencing reads into each species, and as a result very accurate assignment was achieved. However probabilistic approaches such as Expectation-Maximization algorithm [16] could improve classification performance further.

**Table 1:** Summary statistics of RNA-Seq for human and mouse cell lines analyzed in this study

Sample	Species	Cell type	Cell line	Total^a^	PF^b^
ExpID-112	Human	PDAC^c^	PANC-1	62106630	43070936
ExpID-114	Human	PDAC	PK-8	58769786	44701524
ExpID-115	Human	PDAC	PK-9	60832395	44822240
ExpID-116	Human	PDAC	PK-45H	31898332	29286400
ExpID-117	Human	PDAC	PK-45P	35500162	32211253
ExpID-118	Human	PDAC	KLM-1	23673045	20778304
ExpID-119	Human	PDAC	MiaPaca-2	15255557	13715276
ExpID-120	Human	PDAC	Capan-1	41776004	35857527
ExpID-121	Human	PDAC	HOPE	21063312	19314964
ExpID-128	Mouse	Endothelial	SVEC4-10	32128404	29780901
ExpID-129	Mouse	Endothelial	IP-1B	34391406	31548417

^a^Total Number of reads

^b^Number of reads passing Illumina’s filter

^c^Pancreas ductal adenocarcinoma

**Table 2:** Effect of mappable regions between human and mouse on estimated gene expression levels

Sample	Species	Cell line	Human^a^	Mouse^b^	Error (%)^c^	Correlation^d^
ExpID-112	Human	PANC-1	32194053	1821	0.0057	0.9992012
ExpID-114	Human	PK-8	34975755	1867	0.0053	0.9998013
ExpID-115	Human	PK-9	32170808	3484	0.0108	0.9997474
ExpID-116	Human	PK-45H	17278113	1331	0.0077	0.9998448
ExpID-117	Human	PK-45P	18986898	1243	0.0065	0.9997115
ExpID-118	Human	KLM-1	8950786	799	0.0089	0.999563
ExpID-119	Human	MiaPaca-2	2251017	1192	0.0530	0.9997128
ExpID-120	Human	Capan-1	2829135	2195	0.0776	0.9995722
ExpID-121	Human	HOPE	8930382	1110	0.0124	0.9996743
ExpID-128	Mouse	SVEC4-10	1052	18625926	0.0056	0.9987255
ExpID-129	Mouse	IP-1B	6508	19707229	0.0330	0.9988737

^a^Number of reads assigned to human by the CASTIN system

^b^Number of reads assigned to mouse by the CASTIN system

^c^Misassignment rate (%)

^d^Pearson’s correlation coefficient between gene expression levels of human (mouse) cell lines using mappable regions of only human (mouse) and both human and mouse

CASTIN discards reads derived from human or mouse when they were mapped on unmappable (identical sequence between human and mouse) regions, which could result in inaccurate gene expression levels. However, as our algorithm considers “mappable” length instead of transcript length, the effect should be minimal as far as the read coverage within gene is close to uniform after removing the effect of distance from polyA tail and regional GC content. To investigate the effect, gene expression levels of human cell lines using mappable regions of only human and both human and mouse were compared. Gene expression levels of mouse cell lines were also evaluated in a same manner. Table 2 shows the Pearson correlation between the two conditions for each sample. Very strong correlations in both human and mouse indicate that correction of the effect of mappable length worked well.

Additionally, we have performed RNA-seq analysis of artificial mixtures of human (PANC-1 cell line) and mouse (SVEC4-10 cell line) total RNA to evaluate the reproducibility of gene expression quantitation under various tumor-stromal ratios (human content: 0, 25, 50, 75, and 100 %) (Additional file 4: Table S2, Additional file 5: Figure S3). In both human and mouse, the estimated gene expression levels had very high correlation (human; 0.97-0.99, mouse; 0.94-0.99) on the identical line regardless of human-to-mouse ratios. Because mixed RNA samples were sequenced with Illumina GAIIx and pure human or mouse samples were sequenced with HiSeq2000, the correlations slightly decreased when comparing the results from different sequencing platforms. Nonetheless, these results demonstrated the highly reliable and reproducible gene expression quantitation under various conditions with different tumor-stromal ratios.

Finally, we have compared the results obtained from CASTIN with protein expression determined by immunohistochemistry. We applied CASTIN to the dataset of pancreas ductal adenocarcinoma (PDAC) consisting of 8 xenograft samples from different PDAC cell lines (Table 3). We have selected FABP5/Fabp5 gene for analysis because it showed various gene expression ratios between human and mouse in PDAC xenograft samples (human-to-mouse ratios ranged from 0.028 to 3.4, Additional file 6: Figure S4a), and also the antibody with human/mouse cross-reactivity and FFPE compatibility was commercially available. FABP5/Fabp5 stained cancer cells homogeneously except for KLM-1 (Additional file 6: Figure S4b); stromal cells were stained homogeneously or heterogeneously in each sample, reflecting the different cell populations that comprise stroma (e.g. fibroblasts, leukocytes, vascular endothelial cells). Although immunohistochemistry is not strictly quantitative, the relative staining intensity between human cancer and mouse stromal cells well reflected the human to mouse ratio of RNA-Seq reads from the same xenograft tumor.

**Table 3:** Summary statistics of RNA-Seq for PDAC xenograft models analyzed in this study

Sample	Cell line	Total^a^	PF^b^	Human^c^	Mouse^d^	Mouse (%)^e^
ExpID-88	KLM-1	40315040	36210281	14306947	915402	6.01
ExpID-89	Capan-1	42088225	37433177	18147400	2041946	10.11
ExpID-90	PANC-1	42912362	38177843	18700371	2007413	9.69
ExpID-91	PK-1	36841310	33534549	14071808	5663160	28.70
ExpID-92	PK-8	37239118	33971461	15024350	2902408	16.19
ExpID-93	PK-45P	38612205	35070083	11713564	9796740	45.54
ExpID-94	PK-9	40328852	36514269	16929209	2015939	10.64
ExpID-95	MiaPaca-2	39641561	35751983	20439739	2037175	9.06

^a^Total Number of reads

^b^Number of reads passing Illumina’s filter

^c^Number of reads assigned to human by the CASTIN system

^d^Number of reads assigned to mouse by the CASTIN system

^e^Percentage of mouse reads, expressed by d/(c + d)

### Interactome profiles correlate to histology of pancreatic cancer

To demonstrate that interactome profiles of CASTIN correlate histology of cancer and stroma, we applied CASTIN to the dataset of PDAC consisting of 8 xenograft samples from cell lines (Table 3). PDAC was chosen because one of its defining features is the presence of extensive desmoplasia and recent studies have shown that cancer-stromal interaction plays a key role in PDAC development [21].

**Fig. 3:**
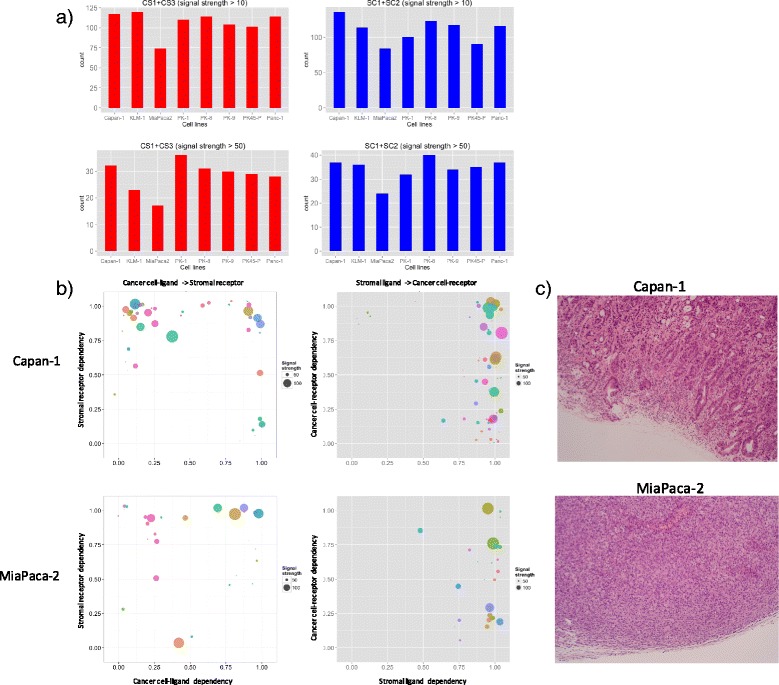
Relationships between interactome profiles and desmoplastic reaction in the PDAC samples. **a** The number of mutually dependent interactions with strong signals in 8 PDAC samples. top left: signal strength >10 in C-S direction, top right: signal strength >10 in S-C direction, bottom left: signal strength >50 in C-S direction, bottom right: signal strength >50 in S-C direction. **b** Interactome profiles of PDAC samples obtained from mice models transplanted with Capan-1 (*top*), which has the strongest mutually dependent interactions, or MiaPaca-2 (*bottom*), which has the weakest mutually dependent interactions, transplanted mouse models. Left: C-S direction, right: S-C direction. **c** Hematoxylin and eosin (H&E) staining of formalin-fixed, paraffin-embedded PDAC tissue obtained from Capan-1 (*top*) and MiaPaca-2 (*bottom*) xenograft mouse models

We hypothesized that the stronger the desmoplastic reaction is, the stronger the signals in zones CS1 (cancer to stroma), CS3 (stroma to stroma), SC1 (stroma to cancer), or SC2 (stroma to stroma) will be. Thus we counted the number of interactions in zone CS1 or CS3, and SC1 or SC2 (Fig. 3a). It clearly showed that Miapaca-2 has weaker signals related to stroma in both directions compared to others such as Capan-1. Such tendency can also be easily seen visually in interactome profiles (Fig. 3b). As expected, Capan-1 shows desmoplastic histology with rich stroma, whereas Miapaca-2 shows medullary histology with poor stroma content (Fig. 3c), which is atypical in PDAC.

These results demonstrate that the global interactome profile reflects the actual cancer-stromal interaction in vivo well, and CASTIN is useful to characterize cancers with respect to cancer-stromal relationships.

### CASTIN detects known interactions targeted by existing drugs and less-characterized druggable interactions

**Fig. 4:**
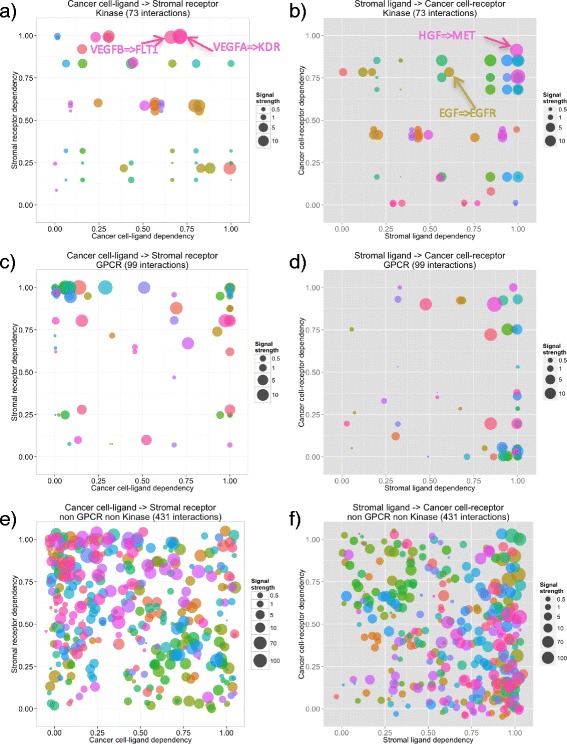
Interactome profiles of PDAC samples. Interactome profiles of kinases in (**a**) C-S direction and (**b**) S-C direction, GPCR in (**c**) C-S direction and (**d**) S-C direction, non-GPCR, non-Kinase in (**e**) C-S direction, and (**f**) S-C direction. Interactions targeted by molecular targeted drugs on the market are indicated by arrows

Next we have investigated profiles of interactions targeted by existing drugs. In particular, we focused on the interactions involving kinases because kinase inhibitors are currently the most successful molecular targeted drugs [22]. PDAC dataset used in the previous section was also analyzed in this section. To summarize eight interactome profiles into a single profile, each averaged evaluation index for every interaction was used (Fig. 4). As shown in Fig. 4a and b, interactions inhibited by currently available molecular targeted cancer drugs such as HGF^1^ -MET^2^ (e.g. Tivantinib), EGF^3^-EGFR^4^ (e.g. Erlotinib), VEGFA^5^-KDR^6^(e.g. Ramucirumab) and VEGFB^7^-FLT1^8^(e.g. Pazopanib) tend to have strong signals and reside around zones SC1 and CS1. While the receptors of the former two interactions (MET and EGFR) are mainly expressed in cancer cells [23, 24] and promote cancer cell proliferation, the receptors of the latter two interactions (KDR and FLT1) are mainly expressed in vascular endothelial cells in microenvironment [25], and drugs targeting these molecules inhibit cancer neovascularization. Based on the signal direction trend of these successfully marketed drugs, the interactions in zones SC1 and CS1 or “mutually dependent” interactions are suggested to be prioritized targets of therapeutic intervention.

Hedgehog signaling between cancer and stroma is known to induce desmoplastic reaction in stroma, and the effect of its inhibition is applied in clinical trials [26] as the improvement of anti-cancer drug delivery was observed in PDAC mouse model [5]. Three hedgehog-related ligand-receptor interactions are included in our database: sonic hedgehog (SHH)– patched 1 (PTCH1), indian hedgehog (IHH)-PTCH1, and IHH–patched 2 (PTCH2). In our analysis, all 3 hedgehog-related interactions were found in zone CS1 with strong signals in Capan-1 xenograft mouse (Additional file 7: Figure S5), suggesting the importance of this signal in pancreatic cancer. However, the signal strength and direction are highly variable between the 8 pancreatic cancer cells. It seems that that the contribution of hedgehog signaling differs among each PDAC sample and that even though hedgehog inhibitor is ineffective for pancreatic cancer as a total [26], personalized medicine may be achieved by developing companion diagnostics to stratify patients based on the contribution of hedgehog signaling in their cancer tissues. Based on the results above, we have searched for less-characterized druggable interactions, which have strong signals and reside in zone CS1 or SC1, in PDAC data. Mutually dependent interactions were extracted in both directions from PDAC data, and interactions with ligand dependency >0.75, receptor dependency >0.75, and signal strength >50 were selected. A literature survey of these interactions were summarized in Table 4.

**Table 4:** Mutually dependent interactions with strong signals in PDAC dataset

ligand	receptor	direction	signal strength^a^	possible relevance for cancer-stromal interactions
SEMA3C	NRP1	C-S	101.5	SEMA3C induces growth and migration of endothelial cells [29], suggesting its angiogenic role; NRP1 also causes desmoplastic reaction in cancer [30].
WNT7B	GPC3	C-S	50	In hepatocellular carcinoma, GPC3 promotes cancer cell growth by Wnt signaling including WNT7B [53].
COL1A2	CD44	S-C	753	CD44 expressed in PDAC regulates its invasion [54].
COL1A1	CD44	S-C	668.4	CD44 expressed in PDAC regulates its invasion [54].
FN1	ITGA3	S-C	496.6	Not reported
COL1A2	ITGA2	S-C	348.4	α2β1 integrin-mediated adhesion on type I collagen promotes the malignant phenotype in PDAC [32].
COL1A1	ITGA2	S-C	341.1	α2β1 integrin-mediated adhesion on type I collagen promotes the malignant phenotype in PDAC [32].
FN1	ITGB6	S-C	180.8	Promotes breast cancer invasion [55].
SEMA4D	PLXNB1	S-C	63.4	Promotes cancer cell motility in PDAC [33].
SFRP1	FZD6	S-C	62	FZD6 overexpressed in several cancers [56]; SFRP1 is a Wnt antagonist.
IGF1	IGF1R	S-C	54.5	IGF1R induces PDAC growth and metastasis [57].

^a^C-S: signal transduction from cancer cell-ligand to stromal receptor

S-C: signal transduction from stromal ligand to cancer cell-receptor

In C-S direction, semapholin 3C (SEMA3C) - neuropilin-1 (NRP1) interaction (Additional file 8: Figure S6a) has the strongest signal. NRP1 is a transmembrane receptor for SEMA3C and vascular endothelial cell growth factor 165 (VEGF165) [27]. NRP1 promotes angiogenesis through binding to VEGF, but mediates antiangiogenic effects by interacting with semapholin 3B and 3 F, the other class 3 semaphorins, indicating that they act as the antagonists that block NRP1 binding to VEGF [28]. Meanwhile, SEMA3C was found to induce growth and migration of endothelial cells [29], which suggests its angiogenic role in cancer. Interestingly, NRP1 also causes desmoplastic reaction in cancer; genetic depletion or antibody neutralization of NRP1 from stromal myofibroblast was shown to reduce cancer growth and fibronectin fibril assembly in vivo [30], although the ligand of NRP1 was not investigated in the report. Considering pronounced desmoplasia of PDAC, our data suggests that NRP1 may contribute to desmoplastic reaction in PDAC.

In S-C direction, interactions including collagens and fibronectins have strong signals. This is reasonable because stromal cells (e.g. fibroblast) highly express extracellular matrix molecules, such as collagen, fibronectin, and laminin [31]. A notable example among these interactions is the one between type I collagen (COL1A1 and COL1A2) and alpha-2 integrin (ITGA2). α2β1 integrin-mediated adhesion on type I collagen has been reported to promote the malignant phenotype in PDAC [32].

Other than collagens and fibronectins, semapholin 4D (SEMA4D) and plexin B1 (PLXNB1) interaction has the strongest signal in zone SC1 (Additional file 8: Figure S6 b). It has been reported that binding of SEMA4D to PLXNB1 promotes cancer cell motility in PDAC [33]. Additionally, increased expression of both SEMA4D and PLXNB1 was associated with poor prognosis [34]. Immunohistochemical analysis showed that SEMA4D was predominantly expressed in the cancer stroma and PLXNB1 was predominantly expressed in cancer epithelial cells in PDAC [34], which is compatible with the evaluation indices at transcript level in our study.

Previous studies have suggested the importance of semapholin signaling in cancer-microenvironment [29, 33–35]. However, its relative importance among all the cancer-stromal interactions has not been quantitatively evaluated due to the lack of methods. Our interactome analysis using CASTIN suggested that semapholin signaling, especially SEMA4D and SEMA3C, plays particularly important roles and these molecules/proteins are potential targets in pancreatic cancer.

Although many researchers have investigated various cancer-stromal interactions which are potential therapeutic targets, prioritization among multiple interactions have not been done as these interactions have been evaluated only individually in most cases. One of the advantages of CASTIN is that using three evaluation indices we can compare multiple interactions based on their role in cancer-stromal interactions and identify which interactions are vital and should be inhibited for clinical approach. Importantly, our ligand-receptor database contains interactions involving extracellular and cell surface proteins, which are easily accessible by biomolecular drugs (large molecules such as antibodies). It is well known that biomolecular drugs greatly expands target interactions/proteins outside of classical druggable proteins, that could be targeted by small molecule drugs. In our study, more than half (431/628) of the interactions identified by CASTIN do not involve kinases or GPCRs, which are typical “druggable” proteins. (Fig. 4e, f).

### Contribution of “functional modules” in cancer-stromal interactome

**Fig. 5:**
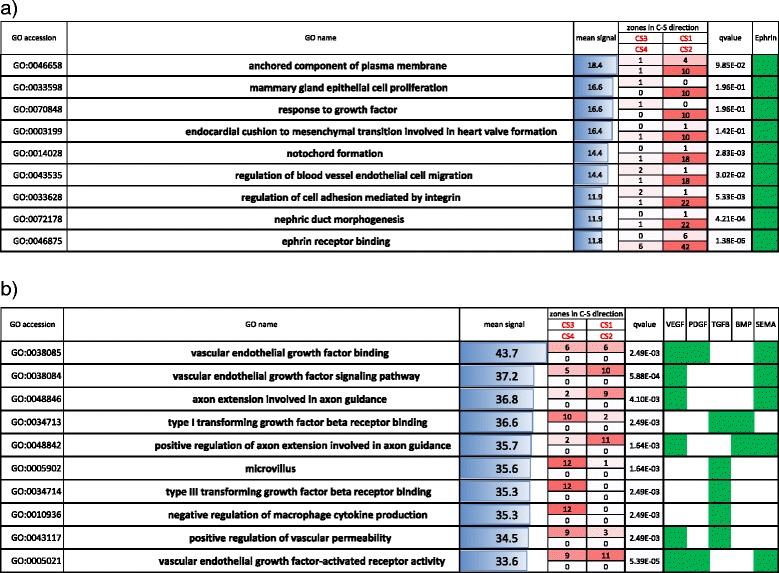
GO categories predominantly located in CS1, CS2 and CS3. **a** Top 9 GO categories predominantly located in CS1 and CS2. 10^th^ GO category was removed because its mean signal strength was < 10. **b** Top 10 GO categories were predominantly located in CS1 and CS3. GO categories were sorted by the percentage of interactions located in the corresponding zones, and then sorted by the mean signal intensity of all the interactions in the GO category. The number of interactions in each zone (CS1, CS2, CS3, and CS4) is shown in ‘zones in C-S direction’ and the intensity of red color is proportional to the number of interactions. False discovery rate (*q*-value) is shown in ‘qvalue’. Representative genes or gene families, which appear in at least 2 GO categories and signal strength >10 for at least 1 interaction, is depicted by green boxes. ‘Mean signal’ refers to the mean signal strength of the interactions in the GO category. Four boxes in ‘zones in C-S direction’ indicate the number of interactions within each zone. Zones CS1 (top right), CS2 (bottom right), CS3 (top left), and CS4 (bottom left)

**Fig. 6:**
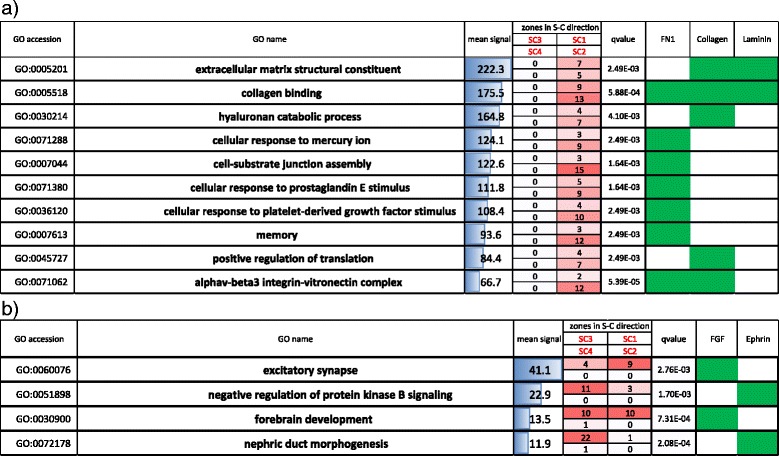
GO categories predominantly located in SC1, SC2 and SC3. **a** Top 10 GO categories predominantly located in SC1 and SC2. **b** Top 4 GO categories predominantly located in SC1 and SC3. Fifth to 10^th^ GO categories were removed because their mean signal strength was <10. Data are processed and presented as in Fig. 5. Four boxes in ‘zones in S-C direction’ indicate the number of interactions within each zone. Zones SC1 (*top right*), SC2 (*bottom right*), SC3 (*top left*), and SC4 (*bottom left*)

CASTIN assigns each ligand-receptor interaction into zones which reflect the direction of signal transduction in cancer-stromal relationships. As genes with similar function are expected to behave similarly, analyzing interactions having similar functions collectively using CASTIN will help us to understand the role of “functional modules” in cancer-stromal relationships. Hence we have investigated the interactome profiles of genes with various molecular functions. Here again we applied CASTIN to the PDAC and the averaged interactome profile was analyzed. We defined an interaction having molecular function when either ligand or receptor in the interaction belonged to Gene Ontology (GO) functional categories. Functional modules with characteristic pattern among 44253 GO categories were shown in Figs. 5 and 6.

**Fig. 7:**
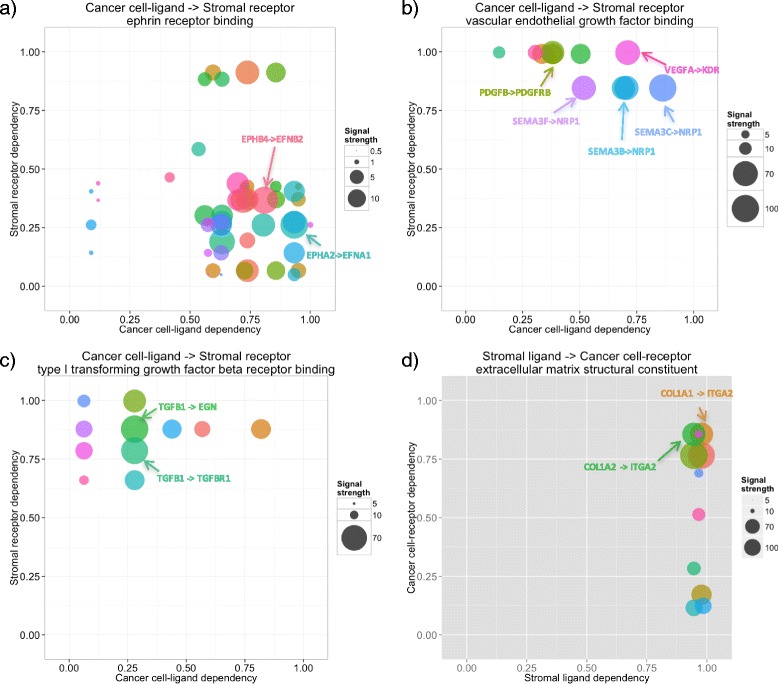
Interactome profiles of PDAC samples for various gene functional categories. **a** Ephrin receptor binding, (**b**) Vascular endothelial growth factor binding, (**c**) Type I transforming growth factor beta receptor binding and (**d**) Extracellular matrix structural constituent. Arrows highlight interactions referred to in the main text

Functional modules located in zone CS1 and CS2 indicate that only cancer cells secrete ligands (Fig. 7a). Actually, however, most interactions in this type are predominantly located in CS2, which indicates auto-regulation of cancer cells. Interestingly, all the functional modules are related to ephrins and Eph receptors. The most representative example is ‘ephrin receptor binding’ (Fig. 7a). Eph-ephrin complexes produce bidirectional signals and affect cancer growth, invasiveness and metastasis [36]. For example, EPHA2 and EPHB4 within ephrin family are widely expressed in cancer cells, and their expression has been linked to cancer progression [36]. Downregulation of EPHA2 or EPHB4 expression with siRNAs or antisense oligonucleotides results in inhibition of malignant cell behavior in culture and cancer growth in vivo [36]. This is in line with our result that both EPHB4-EFNB2 and EPHA2-EFNA1 interactions have strong signals (Fig. 7a).

Functions located in zone CS1 and CS3 are clustered into two groups: functions related to vascular endothelial growth factor (VEGF), platelet-derived growth factor (PDGF), and semaphorins, and functions related to transforming growth factor beta (TGFβ). The VEGF and PDGF related interactions preferentially located in zone CS1 and CS3, which is indicative of stromal cells receiving strong signal from cancer or microenvironment (Fig. 7b). Meanwhile, the TGFβ related interactions predominantly located in zone CS3, which is indicative of microenvironment autoregulation. VEGF and PDGF signaling contribute to angiogenesis in PDAC [37], and VEGF expression in cancer and PDGF receptor expression in stroma are associated with poor prognosis in several types of cancers including PDAC [38, 39]. Semaphorins are important regulators in cancer cells [35], and high expression of SEMA4D was associated with poor survival in PDAC [34] as mentioned in the previous section. TGFβ has the ability to induce fibroblast proliferation in PDAC [40], and autocrine TGFβ signaling regulates myofibrogenesis in carcinoma-associated fibroblasts during fibrosis in breast cancer [41], indicating that stromal cells could be a source of TGFβ in other cancer. In particular, our analysis suggested that TGFβ1 and its receptor TGFβ1 receptor 1 (TβR1) produce strong signal (Fig. 7c). Indeed, TGFβ1, by interacting TβR1, directly elicits desmoplastic reaction in pancreatic cancer [42]. Many TβR1 inhibitors have been developed to improve chemopenetration, and among them, SD-208 reduced fibrosis in cancer microenvironment [43]. Another interaction with strong signaling is TGFβ1–endoglin (EGN). Endoglin is a cell-surface glycoprotein and is part of the TGFβ receptor complex [44]. It also has a crucial role in angiogenesis and is abundantly expressed in vascular endothelial cells at sites of active angiogenesis [44]. In pancreatic cancer tissues, endoglin is highly expressed in endothelial cells forming small capillary-like vessels [45].

GO functional modules predominantly located in zone SC1 and SC2 (Fig. 6a) consistently have strong stromal ligand dependencies, indicating that both cancer and stromal cells are receiving strong signal exclusively from stromal cells. We have found that most of them are related to extracellular matrix such as ‘extracellular matrix structural constituent’ (Fig. 7d). A notable example is the interaction between type I collagen (COL1A1 and COL1A2) and alpha-2 integrin (ITGA2), which we referred in the previous section.

Functional modules located in zone SC1 and SC3 have very low signal strength (Fig. 6b). Top 2 categories ‘excitatory synapse’ and ‘negative regulation of protein kinase B signaling’ has relatively strong signals. However, interactions contributing to the strong signals are related to FN1 and laminin, both of which are related to extracellular matrix and predominantly located in SC1. Thus the we do not discuss the functional modules in this category further.

As shown above, functional modules preferentially located in each zone have distinctive profile reflecting the role in cancer-stromal interactions. These results demonstrate that our interactome analysis reflects molecular functions and useful to prioritize important interactions between cancer and stroma.

## Conclusions

We have developed CASTIN that can quantitatively assess cancer-stromal interactome using RNA-Seq data from cancer xenograft mouse models. Key aspects of CASTIN are high quality, manually curated ligand-receptor database and three evaluation indices, which are easy to interpret and also suitable for visualization. By showing some examples using PDAC dataset, we have shown that these unique features provide researchers with useful information for interpreting cancer-stromal interactome such as a role of each interaction in signal transduction between cancer and stromal cells thus enables prioritization of drug target, and characterization of individual cancer sample in terms of cancer-microenvironment interactions. So far, there are no comparative methods that can perform comprehensive analyses like CASTIN. We have also made the CASTIN software and its viewer publicly available. The software accepts FASTQ (single-end and paired-end) files of RNA-Seq from xenograft samples.

The CASTIN method could also be used to analyze xenograft models of other human cancer types. In the future this method might even be used to identify the cancer-stroma interactome in PDX models and to apply personalized medicine to each patient depending on the many interactions identified. We note that CASTIN is not applicable to early passage PDX models in which human stroma can still be detected, as it would lead to stromal contamination in estimated cancer gene expression levels.

There are several limitations in CASTIN. First, some ligand-receptor relationships may have been left out from our interaction database as our curation process and the database (KEGG or HPRD) only included relationships with adequate experimental evidences. Newly reported protein interactions with sufficient evidence will be included in our ligand-receptor database through continuous updating. Second, it is known that cross species reactivity varies depending on each ligand-receptor interaction [46, 47], which potentially leads to false findings. Detailed information regarding such cross-species interactions is needed in the future. Also, human xenograft models usually involve the use of immunodeficient mice, which have greatly reduced number of lymphocytes. Therefore, the interactions between cancer cell and such lymphocytes, which are possibly druggable, will not be covered by the CASTIN analysis.

Despite the above limitations, currently there are no comparable bioinformatics methods, that can perform comprehensive and quantitative analysis of cancer-stroma interactome like CASTIN. It is hopefully expected that CASTIN will accelerate researchers’ understanding of the whole picture of cancer-stromal interactome quantitatively and visually, and discover critical interactions that are clinically relevant but couldn’t be discovered by sample cancer sequencing analysis so far.

## Methods

### Cell culture

PDAC cell lines KLM-1, MiaPaca-2, PANC-1, PK-8, PK-45P and PK-45H were purchased from RIKEN Bio-Resource Center (Saitama, Japan), PK-1 and PK-9 were purchased from Tohoku University Cell Resource Center (Sendai, Japan), and Capan-1 was purchased from American Type Culture Collection (ATCC, Manassas, USA). KLM-1, Panc-1, PK-1, PK-8, PK-45P, PK-9, PK-45H cells were cultured in RPMI1640 (WAKO Pure Chemical Industries, Osaka, Japan) supplemented with 10 % FBS and 100 mg/ml penicillin/streptomycin (WAKO Pure Chemical Industries, Osaka, Japan). Capan-1 was cultured in DMEM (WAKO Pure Chemical Industries, Osaka, Japan) supplemented with 20 % FBS, 100 mg/ml penicillin/streptomycin and 200 mmol/L L-Alanyl-L-Glutamine (WAKO Pure Chemical Industries, Osaka, Japan). MiaPaca-2 was cultured in DMEM supplemented with 10 % FBS and 100 mg/ml penicillin/streptomycin.

### Animal study

Five to six weeks old BALB/cAJcl-nu/nu female nude mice (CLEA Japan, Tokyo) were used as the host for cancer xenograft model. Briefly, 5 × 10^6^ cells were suspended in 100 μl of phosphate buffer saline (PBS (−)), and were injected subcutaneously into the right flank of mice. The animals were sacrificed when the diameter of tumor reached 5 mm.

For immunohistochemistry, samples were formalin-fixed and embedded in paraffin, and then cut into 7-μm thick serial sections and mounted onto microscope slides.

### Transcriptome sequencing of xenograft and cell line samples

Tumors resected from the mice were frozen and the suspended in trizol reagent (Thermo Fisher Scientific Inc, Waltham, USA), and total RNA was extracted according to the manufacturer’s instruction. Cultured cells were suspended in trizol reagent (Thermo Fisher Scientific Inc, Waltham, USA), and total RNA was extracted according to the manufacturer’s instruction. One microgram of total RNA was used as the starting material for a 50-bp paired-end transcriptome-sequencing protocol using an Illumina GAIIx sequencer (Illumina, San Diego, CA, USA). Briefly, PolyA+ RNA was purified from total RNA and fragmented using divalent cations. RNA quality as assessed by RNA integrity number (RIN) using a bioanalyzer (Agilent), gave a median RIN of 9.0 (ranged from 6.1 to 10). Double stranded cDNA was synthesized using SuperScript II Reverse Transcriptase (Invitrogen), and its overhang was converted into blunt end using T4 DNA polymerase. 3’ end of the blunt end was adenylated by Klenow fragment, and PE adapter was ligated. Without size selection, the cDNA library was amplified using PCR. For PCR amplification, 1ul of PCR primer PE 1.0 and 2.0, and 0.5 μL of Phusion DNA polymerase (Finnzymes Oy) were used in a final volume of 50 μL. The PCR condition was as follows: 98 °C for 5 min, then 15 cycles of 98 °C for 10 s, 65 °C for 30 s, and 72 °C for 30 s, followed by 72 °C for 5 min before cooling to 4 °C. PCR primers were removed by QIA quick PCR Purification Kit. Each library was loaded into its own single Illumina flow cell lane, producing 50-mer paired-end reads for each sample. The raw sequences have been deposited in the DDBJ Sequence Read Archive under accession number DRA004736.

### Transcriptome sequencing of mixture of human and mouse cell lines

Total RNA of each cell line was extracted and RNA quality was assessed by RIN as described in the previous section. Total RNA from cell lines PANC-1 (human) and SVEC4-10 (mouse) was mixed at the ratios of 1:3, 1:1, and 3:1 ratio. The amount of total RNA was Assayed in the Qubit RNA assay kit (Thermo Fisher Scientific Inc, Waltham, USA).

One microgram of total RNA was used as the starting material for the preparation of transcriptome-sequencing library using TruSeq stranded mRNA library preparation kit (Illumina, San Diego, CA, USA) following the manufacturer’s directions. Libraries were sequenced 100 bp paired-end on Hiseq2000 sequencer (Illumina). Four libraries were loaded into single lane of Illumina flow cell, producing more than 30 million paired-end reads for each sample. Only the first 50 bp of each paired-end was used for the analysis to compare gene expression levels with the samples sequenced with Illumina GAIIx.

### Read mapping and differential taxonomy assignment

Paired-end reads were aligned to all RefSeq transcripts of human (hg19 coordinates) and mouse (mm10 coordinates) allowing up to one mismatch. Alignments were performed by using TMAP version 3.4.1 [48] with the –a 2 –s 1 –g 3 –u 50 preset. Paired-end reads were considered as RefSeq transcripts if both ends in the pair were mapped to the same RefSeq transcript and each read in the pair was not mapped to other RefSeq transcripts of a different gene. A pair can be mapped to multiple RefSeq transcripts if the condition was met for multiple splice variants of a same gene. NCBI Gene IDs were used to map RefSeq transcripts to genes. Homologene [49] downloaded from NCBI website was used to convert Gene ID of mouse to that of human. When a single human gene was homologous to multiple mouse genes, sum of the expressions of these mouse genes were used.

### Quantification of gene expression

After the read mapping, we removed biases of gene expression levels derived from gene length, distance from poly-A tail, mappability, and regional GC content. We modeled the count of reads for *j-*th nucleotide of gene *i* using a Poisson linear model:

$$ \mathrm{E}\left[ \log {c}_{ij}\right]= \log \frac{N_i{m}_{ij}}{{\displaystyle {\sum}_k}{m}_{ij}}{v}_i+\alpha {g}_{ij}+\beta {d}_{ij} $$

Where *c*_*ij*_ assumed to follow a Poisson distribution is the count of reads covering the *j*-th nucleotide from poly-A tail of gene *i*, *N*_*i*_ is the length of gene *i*, *m*_*i*,*j*_ is the number of mappable 50 bp covering the *j*-th nucleotide, *v*_*i*_ is the true expression of gene *i*, *v*_*i*,*j*_ is the GC% around 50 bp of the *j*-th nucleotide, *d*_*i*,*j*_ is the distance from poly-A tail, *α* is the coefficient of the effect of GC content, and *β* is the coefficient of the effect of distance from poly-A tail. *α* and *β* depend on experiments, but are independent of genes or nucleotide positions. We assume that all the estimated parameters are identical in human and mouse because sequencing process is the same. 50 bp mappability of each nucleotide was computed using vmatch version 2.0 [50], allowing up to one mismatch. Parameter optimization of the model was performed iteratively as described previously [18]. Initial value of *v*_*i*_ was ${\displaystyle {\sum}_{k=1}^{N_i^{\hbox{'}}}\frac{{\displaystyle {\sum}_{l=1}^{N_i^{\hbox{'}}}}{m}_{il}}{N_i^{\hbox{'}}{m}_{ik}}{c}_{ik}}$, where *N*_*i*_^'^ = min(*N*_*i*_, 3000). *c*_*ij*_ is significantly affected by the bias arising from the distance to poly-A tail when *j* and *N*_*i*_ are large, and thus the convergence would be faster if *N*_*i*_^'^ instead of *N*_*i*_ was used for the initialization. Poisson regression in each iteration was done using a glm function of R environment via rJava interface. In order to reduce computational time while maintaining accuracy of the estimated parameters, only transcripts satisfying the following conditions were used for parameter optimization: (i) no splicing variant existed, (ii) the transcript length was more than 8kbp and (iii) more than 80 % of the transcript was covered with at least 1 read. After parameter optimization, estimated copy number of gene *i* is calculated as follows:

$$ {\overset{\sim }{v_i}}^{{\textstyle \hbox{'}}}=\frac{\overset{\sim }{v_i}}{Z}=\frac{{\displaystyle {\sum}_{k=1}^{N_i}}{c}_{ik}^{{\textstyle \hbox{'}}}}{Z{\displaystyle {\sum}_{k=1}^{N_i}} \exp \left(\alpha {g}_{ik}+\beta {d}_{ik}\right)} $$

where *c*_*ij*_^'^ is the count of reads starting at the *j*-th nucleotide of gene *i* and *Z* is a normalization factor so that sum of ${\overset{\sim }{v_i}}^{{\textstyle \hbox{'}}}$ below the 95th percentile be 300,000, which is roughly the average number of mRNA molecules present in a cell [51]. Note that *c*_*ij*_^'^ instead of *c*_*ij*_ was used in the estimation step because the effect of GC% was expected to be corrected more accurately. Conversely, *c*_*ij*_ was used in the optimization step since *c*_*ij*_^'^ was so sparse that the parameter could not be estimated accurately.

### Indices for interactome data evaluation

For the purpose of quantitative and comprehensive evaluation of interactome, we have introduced three evaluation indices for each signal direction for each gene. We assume that there are *P* pairs of ligand and receptor genes in our in-house database. Let *L*_*Ci*_, *L*_*Si*_, *R*_*Cj*_, and *R*_*Sj*_ be normalized gene expression levels of ligand gene *i* (*i* = 1, …, *P*) of human (cancer), ligand gene *i* (*i* = 1, …, *P*) of mouse (stroma), receptor gene *j* (*j* = 1, …, *P*) of human (cancer), receptor gene *j* (*j* = 1, …, *P*) of mouse (stroma), respectively. We define three evaluation indices, ligand dependency *X*, receptor dependency *Y*, signal strength *Z* for each direction as follows:

- C-S direction
  - $$ {\mathrm{X}}_{\mathrm{C}\to \mathrm{S},i}=\frac{L_{Ci}}{L_{Ci}+{L}_{Si}} $$
  - $$ {\mathrm{Y}}_{\mathrm{C}\to \mathrm{S},j}=\frac{R_{Sj}}{R_{Cj}+{R}_{Sj}} $$
  - $$ {\mathrm{Z}}_{C\to S,i,j}=\sqrt{L_{Ci}\cdot {R}_{Sj}} $$
- S-C direction
  - $$ {\mathrm{X}}_{\mathrm{S}\to \mathrm{C},i}=\frac{L_{Si}}{L_{Ci}+{L}_{Si}} $$
  - $$ {\mathrm{Y}}_{\mathrm{S}\to \mathrm{C},j}=\frac{R_{Cj}}{R_{Cj}+{R}_{Sj}} $$
  - $$ {\mathrm{Z}}_{S\to C,i,j}=\sqrt{L_{Si}\cdot {R}_{Cj}} $$

### In-house ligand-receptor database construction

We have constructed an in-house ligand-receptor database. The database construction consisted of three main steps (i) extraction of localization information from Human Protein Reference Database (HPRD) [20] (ii) extraction of ligand-receptor interaction from Kyoto Encyclopedia of Genes and Genomes (KEGG) data [19] (iii) curation by reviewing original literature.

First, proteins localized primarily to extracellular space and plasma membrane were selected as ligand and receptor candidates, respectively. Information of primary localization was downloaded from Human Protein Reference Database (HPRD, release 8) [20] on 9 September 2009.

Among all the pairs of ligand and receptor candidates, only those appeared in protein-protein interaction in Kyoto Encyclopedia of Genes and Genomes (KEGG) pathway database [19] (release 55.0, downloaded on 7 August 2010) proceeded to the next curation step. Direction of interaction was determined according to relations (activation, inhibition, binding/association, or indirect effect) in KEGG database. For example, if ‘A activates B’ appeared, A and B became candidates of ligand and receptor, respectively. If the relationship was undirectional such as ‘binding/association’, direction was determined at random with one exception: proteins in Ephrin and Ephrin-receptor families appeared in Axon guidance pathway (Entry: hsa04360), were assigned as ligand and receptor, and vice versa [36]. Interactions occurring within the same cell were removed manually.

Finally, researchers in the field of biology curated each interaction by carefully reviewing the original literature attached in the KEGG database.

### Visualization interface of the CASTIN output

We have also developed a web interface for interactive 2D-visualization of the CASTIN output (http://gpatgazeza.tmd.ac.jp/CASTIN_viewer/index.php). Here we introduce a brief description of the interface (Additional file 3: Figure S2). Please refer to the manual for detail.

There are two 2D scatter plots, each of which corresponding to S-C and C-S direction of signals. In each scatter plot, horizontal axis represents ligand dependency and vertical axis represents receptor dependency. Each circle represents ligand-receptor interactions, with its radius proportional to the log of signal strength of the interaction.

When users hover cursor over the circles, gene symbols of the ligand and the receptor and its signal strength is shown.

By inputting the threshold value of signal strength in “Threshold of signal strength” box and pressing “View” button, users can hide weak (and thus possibly non-significant) interactions. Additionally, users can search specific genes by entering gene symbol (s) in “Search Genes (Gene Symbol)” box.

### Gene ontology analysis

We used all the gene ontology categories of human, irrespective of their hierarchies. All gene ontology categories and the genes belonging to each category were retrieved from Gene Ontology Consortium [52] on February 10, 2016.

### Immunohistochemistry

Cut specimens of formalin-fixed and paraffin-embedded mouse tumors (Capan-1, KLM-1, MiaPaCa-2, PANC-1, PK-1, PK-8, PK-9 and PK45-P) were obtained from tumor transplanted mice as described above. After de-paraffinized by Xylene (Wako Pure Chemical Industries, Japan) for 10 min at room temperature, the specimen slides were treated with Citrate buffer (pH 6.0) (Abcam, UK) by an autoclave (TOMY Seiko, Japan) at 121 °C for 5 min in order to retrieve protein antigens. Endogenous peroxidase activity was masked by incubating the slides with 3 % H_2_O_2_ (Sigma Aldrich, USA) for 10 min at room temperature. The slides were incubated with 2 % BSA (Sigma Aldrich) / PBS for 1 h at room temperature to block non-specific protein-antibody reactions. Then the slides were incubated with anti-FABP5 antibody (Rabbit #39926, Cell Signaling Technology, USA) at 1/200 dilution for an over-night at 4 °C. Histostar (MBL, Japan) and DAB solution (MBL) were used to detect the FABP5 1^st^ antibody signals under a microscope (Olympus, Japan) with the nuclear staining with Hematoxylin (Sakura Finetek Japan, Japan).

### Statistical tests

All the *p*-values were calculated by Binomial test (one-sided) and transformed into *q*-values for false discovery rate (FDR) analysis using the ‘qvalue’ package from Bioconductor.

## Additional files

Additional file 1: Figure S1. Read count biases in a xenograft sample from PDAC cell line (PK-1). Randomly chosen 100,000 read count residuals from fitted model are plotted against (a, b) distance from poly-A site and (c, d) regional GC content. Residuals with biases (a, c) and without biases (b, d). Randomly chosen 100,000 points are plotted. Straight line in each plot indicates the bias estimated from 200 genes using Poisson linear model (see Methods). (PDF 70 kb)
Additional file 2: Table S1. Ligand-receptor interactions used in the CASTIN system. (PDF 175 kb)
Additional file 3: Figure S2. Screenshot of the CASTIN interactive viewer. Hover a cursor over a circle and it displays the interacting genes represented and their value of signal strength. (PDF 1376 kb)
Additional file 4: Table S2. Summary statistics of RNA-seq for mixture of total RNA from human (PANC-1) and mouse (SVEC4-10) cell lines. (PDF 37 kb)
Additional file 5: Figure S3. Comparison of gene expression levels of samples with different RNA mixture ratios (PANC-1 and SVEC4-10) estimated by CASTIN. (a) gene expression levels of human (human content: 25 %, 50 %, 75 %, 100 %) and (b) mouse (mouse content: 100 %, 75 %, 50 %, 25 %) after global normalization in each species. On the bottom of the diagonal: the bivariate scatter plots with the identity line. On the top of the diagonal: the value of the cosine correlation. (PDF 608 kb)
Additional file 6: Figure S4. FABP5/Fabp5 expression in RNA-Seq (estimated by CASTIN) and Immunohistochemistry. (a) gene expression levels. (b) Immunohistochemical (IHC) and hematoxylin and eosin (H&E) staining of close sections. Sections of PDAC xenograft cancer derived from each cell line were stained for FABP5/Fabp5 (left) or with H&E (right). The slightly different distribution of tumor and stromal cells between H&E and the corresponding IHC sections was due to the physical distance between the two sections. (PDF 10295 kb)
Additional file 7: Figure S5. Distribution of hedgehog-related interactions in PDAC samples. a) Cancer cell SHH to stromal PTCH1. b) Cancer-cell IHH to stromal PTCH1. c) Cancer-cell IHH to stromal PTCH2. (PDF 162 kb)
Additional file 8: Figure S6. Distribution of mutually dependent interactions with strong signals in PDAC samples. a) Stromal SEMA3C to cancer-cell PLXNB1. b) Cancer-cell SEMA4D to stromal NRP1. (PDF 136 kb)

## Abbreviations

FDR: False discovery rate
GO: Gene ontology
GSEA: Gene set enrichment analysis
HPRD: Human protein reference database
KEGG: Kyoto encyclopedia of genes and genomes
PBS: Phosphate buffer saline
PDAC: Pancreas ductal adenocarcinoma
RIN: RNA integrity number
TPM: Transcripts per kilobase million

## Footnotes

1 Hepatocyte growth factor

2 MET proto-oncogene, receptor typrosine kinase

3 Epidermal growth factor

4 Epidermal growth factor receptor

5 Vascular endothelial growth factor A

6 Kinase insert domain receptor

7 Vascular endothelial growth factor B

8 FMS related tyrosine kinase 1
