# Supplementary figures and images for: CASTIN: a system for comprehensive analysis of cancer-stromal interactome
Source: BMC Genomics. 2016 Nov 9;17:899. doi: 10.1186/s12864-016-3207-z (PMC5103609; doi:10.1186/s12864-016-3207-z)

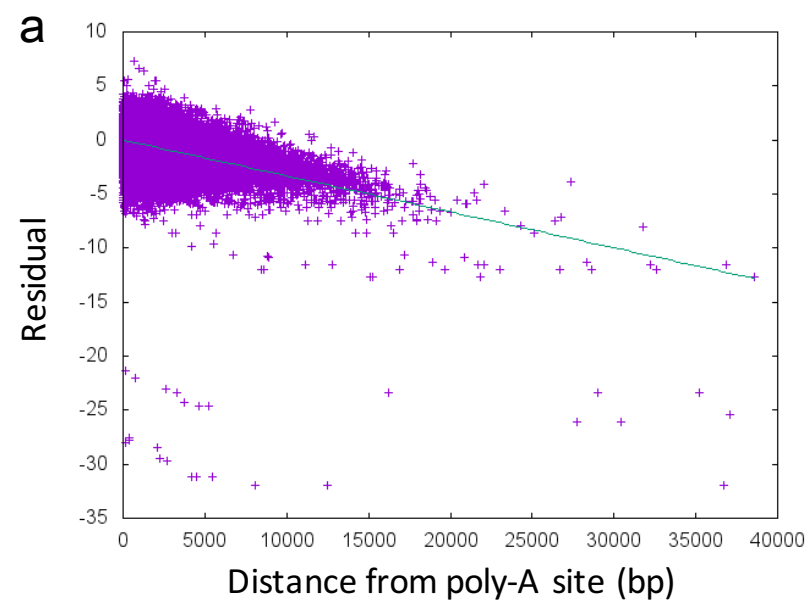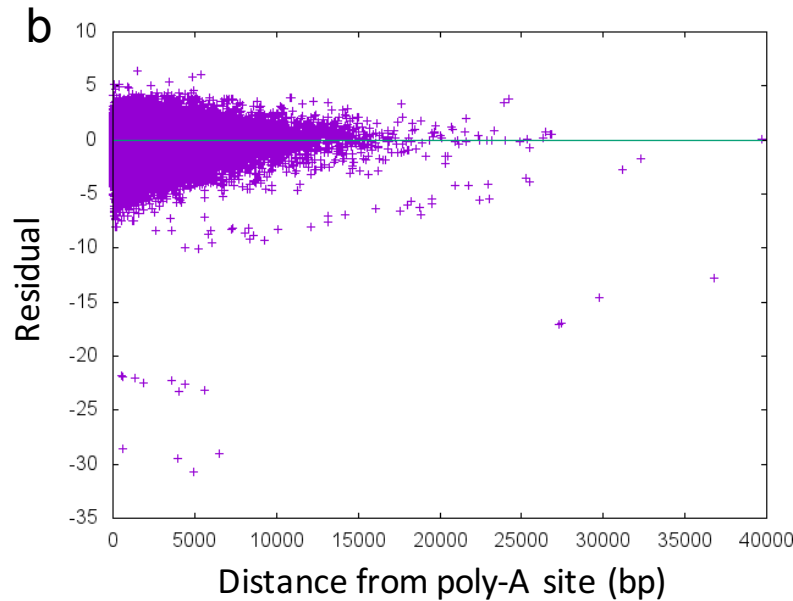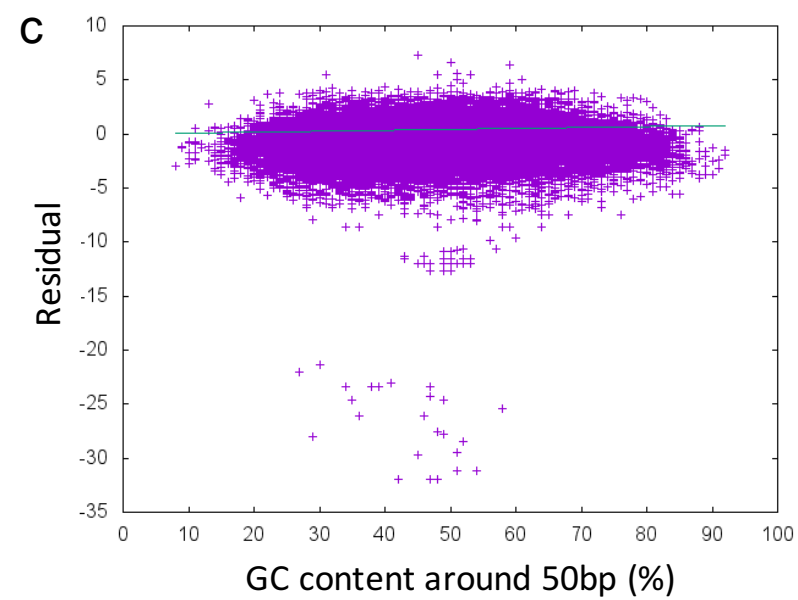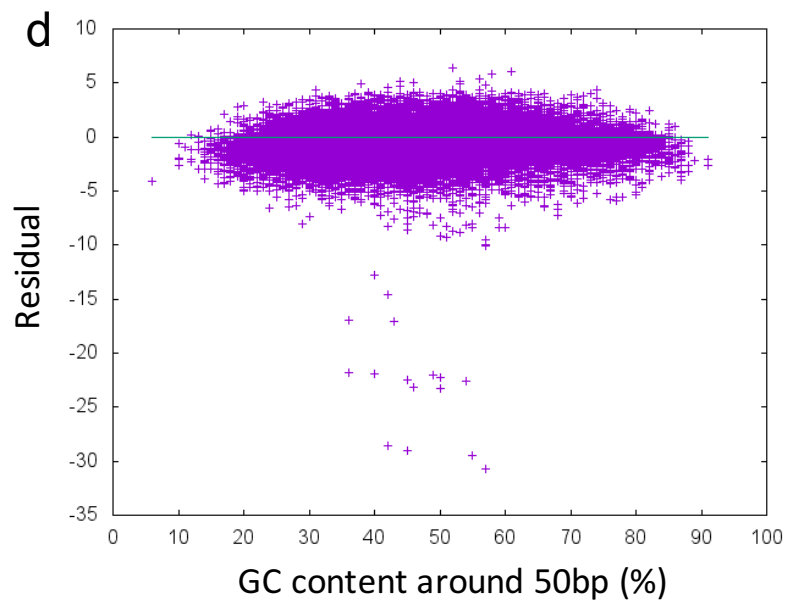

Supplement: Additional file 1: Figure S1. — Read count biases in a xenograft sample from PDAC cell line (PK-1). Randomly chosen 100,000 read count residuals from fitted model are plotted against (a, b) distance from poly-A site and (c, d) regional GC content. Residuals with biases (a, c) and without biases (b, d). Randomly chosen 100,000 points are plotted. Straight line in each plot indicates the bias estimated from 200 genes using Poisson linear model (see Methods). (PDF 70 kb) [file 12864_2016_3207_MOESM1_ESM.pdf]

a

Human (PANC-1)

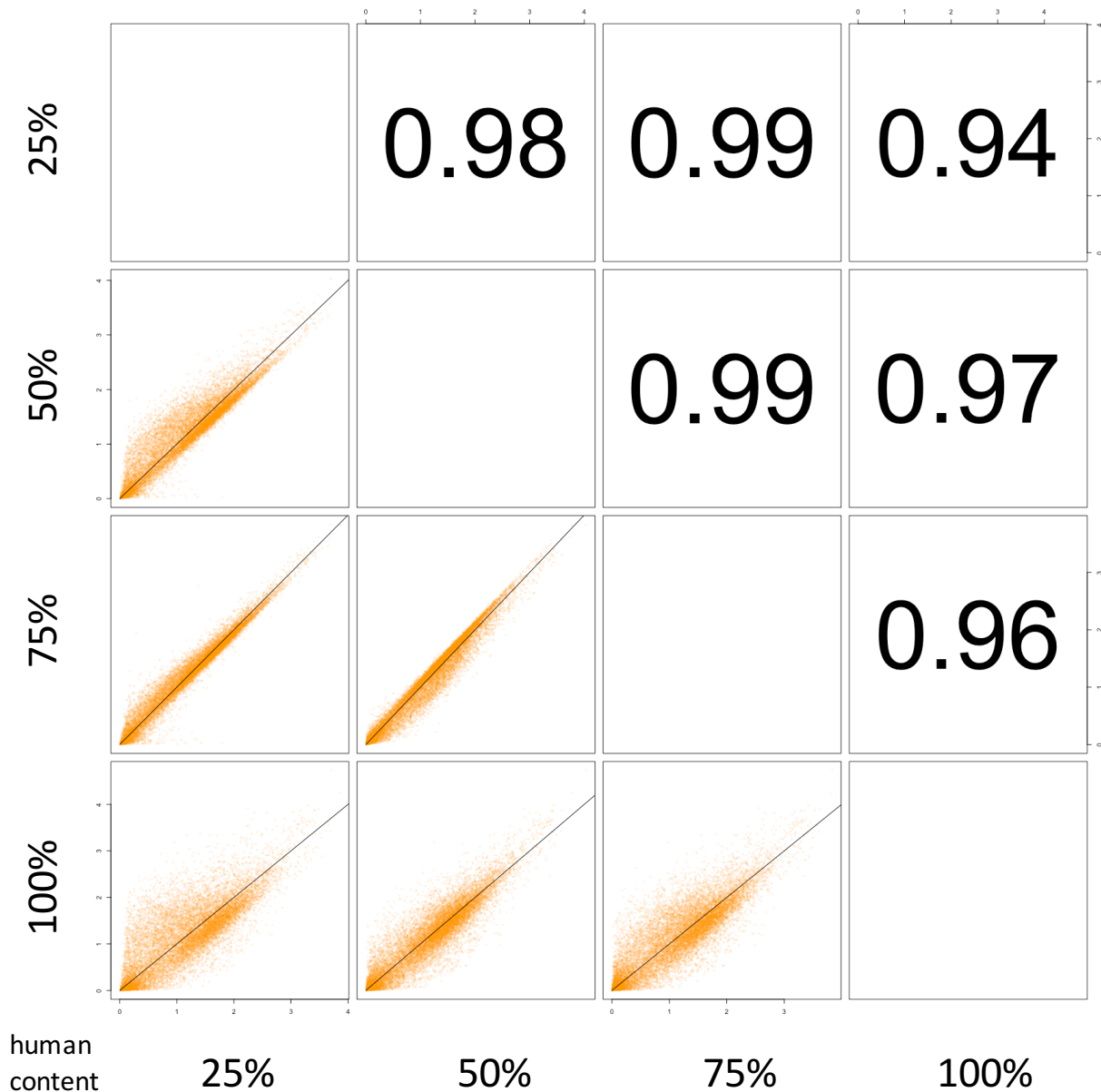

b

Mouse (SVEC4-10)

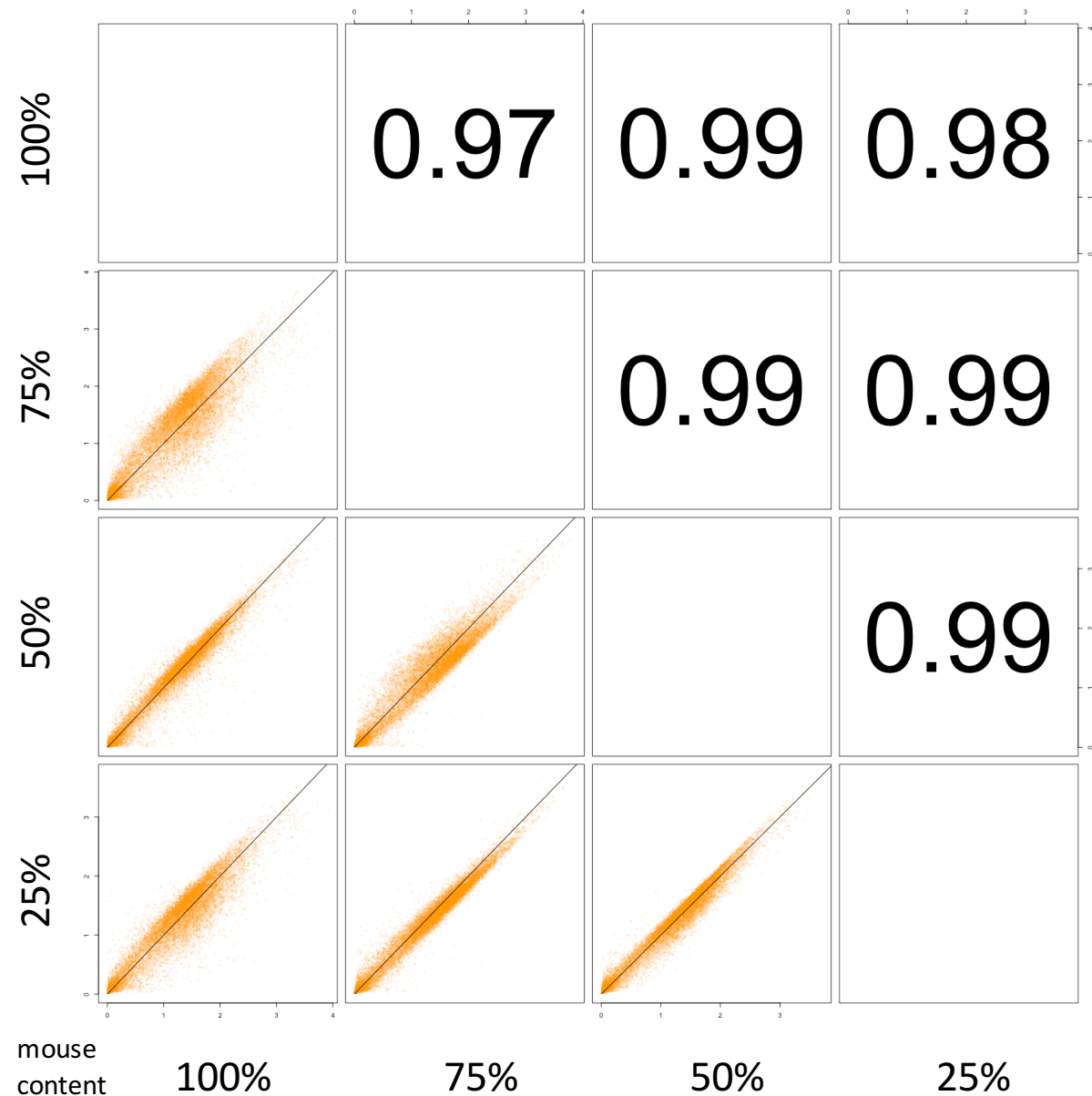

Supplement: Additional file 5: Figure S3. — Comparison of gene expression levels of samples with different RNA mixture ratios (PANC-1 and SVEC4-10) estimated by CASTIN. (a) gene expression levels of human (human content: 25 %, 50 %, 75 %, 100 %) and (b) mouse (mouse content: 100 %, 75 %, 50 %, 25 %) after global normalization in each species. On the bottom of the diagonal: the bivariate scatter plots with the identity line. On the top of the diagonal: the value of the cosine correlation. (PDF 608 kb) [file 12864_2016_3207_MOESM5_ESM.pdf]

FABP5/Fabp5 in RNA-seq

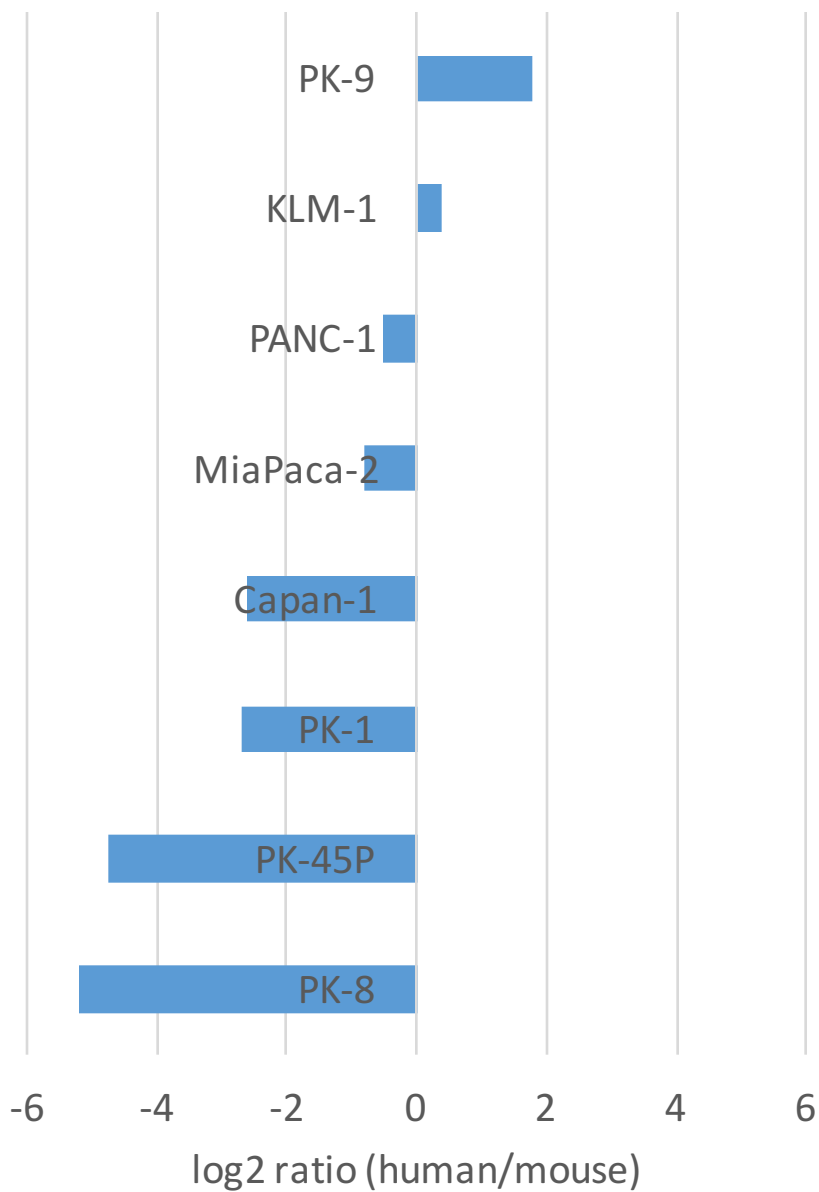

FABP5 IHC

H&E

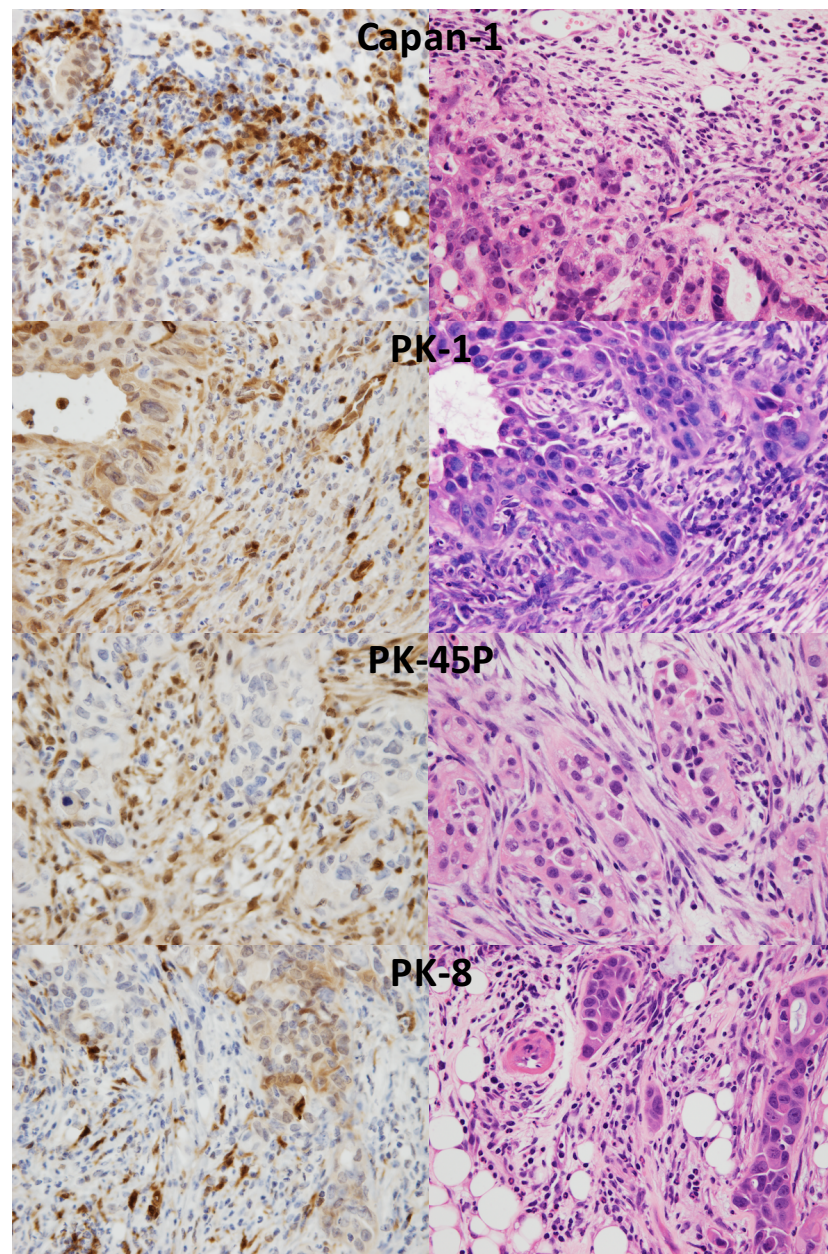

FABP5 IHC

H&E

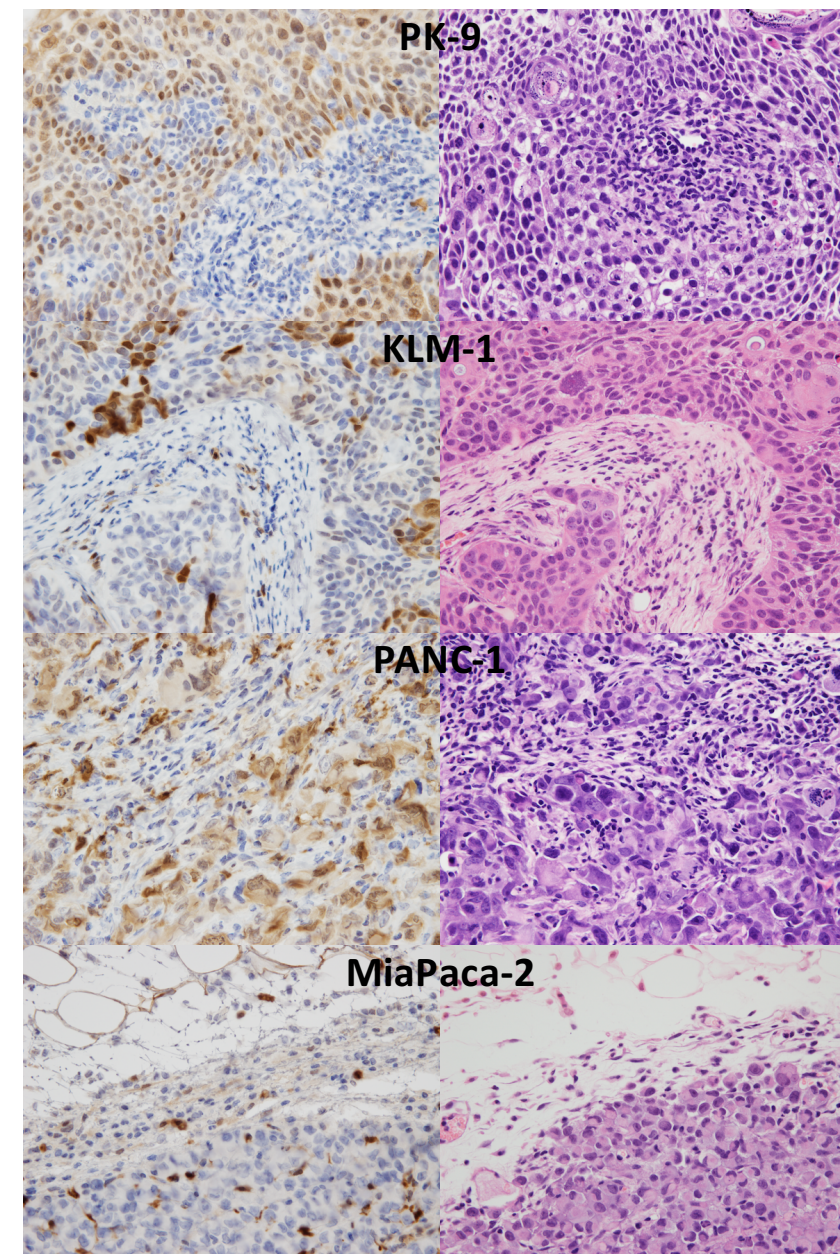

Supplement: Additional file 6: Figure S4. — FABP5/Fabp5 expression in RNA-Seq (estimated by CASTIN) and Immunohistochemistry. (a) gene expression levels. (b) Immunohistochemical (IHC) and hematoxylin and eosin (H&E) staining of close sections. Sections of PDAC xenograft cancer derived from each cell line were stained for FABP5/Fabp5 (left) or with H&E (right). The slightly different distribution of tumor and stromal cells between H&E and the corresponding IHC sections was due to the physical distance between the two sections. (PDF 10295 kb) [file 12864_2016_3207_MOESM6_ESM.pdf]

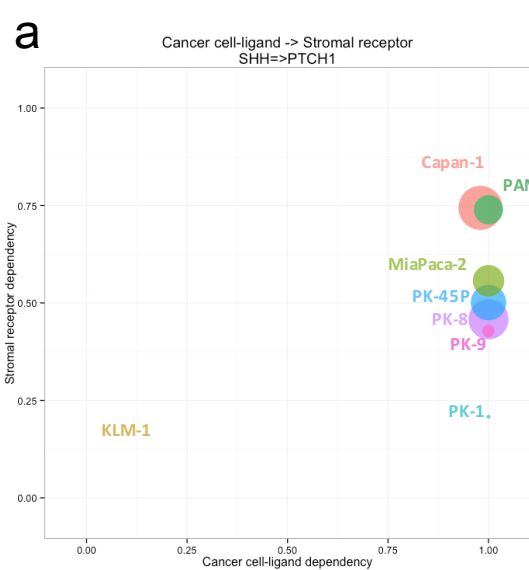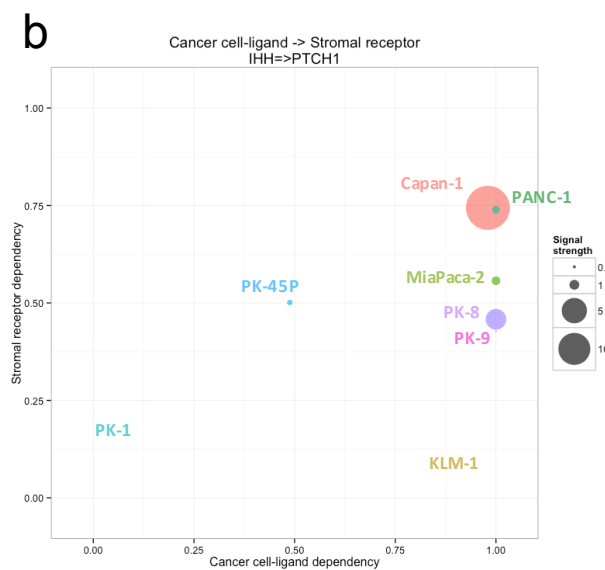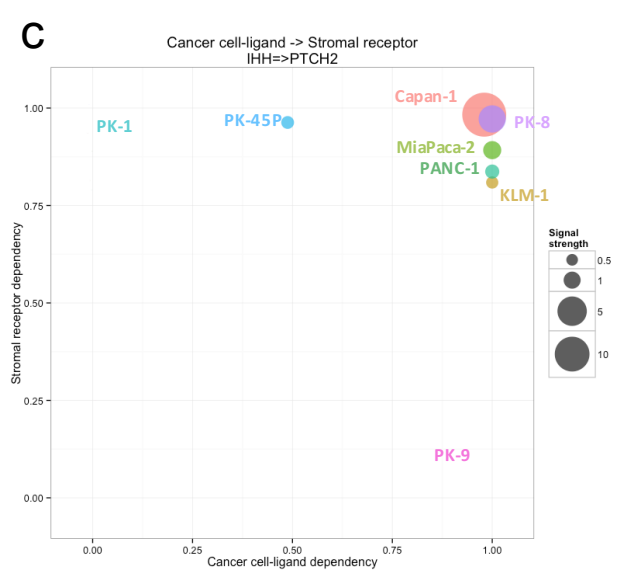

Supplement: Additional file 7: Figure S5. — Distribution of hedgehog-related interactions in PDAC samples. a) Cancer cell SHH to stromal PTCH1. b) Cancer-cell IHH to stromal PTCH1. c) Cancer-cell IHH to stromal PTCH2. (PDF 162 kb) [file 12864_2016_3207_MOESM7_ESM.pdf]
