# Supplementary material for: CASTIN: a system for comprehensive analysis of cancer-stromal interactome
Source: BMC Genomics. 2016 Nov 9;17:899. doi: 10.1186/s12864-016-3207-z (PMC5103609; doi:10.1186/s12864-016-3207-z)
Supplement: Additional file 2: Table S1. — Ligand-receptor interactions used in the CASTIN system. (PDF 175 kb) [file 12864_2016_3207_MOESM2_ESM.pdf]

**Supplementary Table 1. Ligand-receptor interactions used in the CASTIN**

| **ligand** | **receptor** |
|---|---|
| ADAM17 | NOTCH1 |
| ADIPOQ | ADIPOR2 |
| ALCAM | CD6 |
| AMH | AMHR2 |
| AREG | EGFR |
| AVP | AVPR2 |
| BMP2 | ACVR1 |
| BMP2 | BMPR1A |
| BMP2 | BMPR1B |
| BMP2 | BMPR2 |
| BMP4 | BMPR2 |
| BMP7 | ACVR2A |
| BMP7 | BMPR1B |
| BMP7 | BMPR2 |
| BTC | EGFR |
| BTC | ERBB4 |
| C2 | C3 |
| C3 | C3AR1 |
| C3 | C5 |
| C3 | CR1 |
| C3 | CR2 |
| C3 | ITGAM |
| C5 | C5AR1 |
| C5 | C6 |
| CCL1 | CCR8 |
| CCL13 | CCR3 |
| CCL13 | CCR5 |
| CCL14 | CCR1 |
| CCL14 | CCR3 |
| CCL14 | CCR5 |
| CCL15 | CCR1 |
| CCL15 | CCR3 |
| CCL16 | CCR1 |
| CCL16 | CCR5 |
| CCL16 | CCR8 |
| CCL17 | CCR4 |
| CCL17 | CCR8 |
| CCL19 | CCR10 |
| CCL19 | CCR7 |
| CCL2 | CCR1 |
| CCL2 | CCR10 |
| CCL2 | CCR5 |
| CCL20 | CCR6 |

| | |
|---|---|
| CCL21 | CCR7 |
| CCL22 | CCR4 |
| CCL23 | CCR1 |
| CCL24 | CCR3 |
| CCL25 | CCR10 |
| CCL25 | CCR9 |
| CCL26 | CCR1 |
| CCL26 | CCR3 |
| CCL27 | CCR10 |
| CCL28 | CCR10 |
| CCL28 | CCR3 |
| CCL3 | CCR1 |
| CCL3 | CCR3 |
| CCL3 | CCR4 |
| CCL3 | CCR5 |
| CCL3L1 | CCR1 |
| CCL3L1 | CCR3 |
| CCL3L1 | CCR5 |
| CCL3L3 | CCR5 |
| CCL4 | CCR1 |
| CCL4 | CCR3 |
| CCL4 | CCR5 |
| CCL4 | CCR8 |
| CCL5 | CCR1 |
| CCL5 | CCR3 |
| CCL5 | CCR4 |
| CCL5 | CCR5 |
| CCL7 | CCR1 |
| CCL7 | CCR10 |
| CCL7 | CCR3 |
| CCL7 | CCR5 |
| CCL8 | CCR1 |
| CCL8 | CCR3 |
| CCL8 | CCR5 |
| CD22 | PTPRC |
| CD274 | PDCD1 |
| CD86 | CD28 |
| CD48 | CD244 |
| CD58 | CD2 |
| CD70 | CD27 |
| CD80 | CD28 |
| CFH | C3 |
| CFI | C3 |
| CGA | LHCGR |
| CHRD | BMP2 |
| CLCF1 | CNTFR |

| | |
|---|---|
| CLDN3 | CLDN1 |
| CLDN5 | CLDN3 |
| CNTF | CNTFR |
| CNTF | IL6ST |
| CNTF | LIFR |
| CNTN2 | CNTNAP2 |
| CNTN2 | L1CAM |
| CNTNAP1 | NFASC |
| COL1A1 | CD44 |
| COL1A1 | ITGA2 |
| COL1A1 | ITGA5 |
| COL1A2 | CD44 |
| COL1A2 | ITGA2 |
| COL1A2 | ITGA2B |
| COL1A2 | ITGB3 |
| COL2A1 | ITGA2B |
| COL5A1 | SDC3 |
| CRH | CRHR1 |
| CSF1 | CSF1R |
| CSF2 | CSF2RB |
| CSF3 | CSF3R |
| CTF1 | IL6ST |
| CTF1 | LIFR |
| CTSG | F2R |
| CTSG | F2RL1 |
| CTSG | F2RL2 |
| CX3CL1 | CX3CR1 |
| CXCL1 | IL8RA |
| CXCL1 | IL8RB |
| CXCL10 | CCR3 |
| CXCL10 | CXCR3 |
| CXCL11 | CCR3 |
| CXCL11 | CXCR3 |
| CXCL12 | CXCR4 |
| CXCL13 | CCR10 |
| CXCL13 | CXCR3 |
| CXCL13 | CXCR5 |
| CXCL16 | CXCR6 |
| CXCL2 | IL8RA |
| CXCL2 | IL8RB |
| CXCL3 | IL8RA |
| CXCL3 | IL8RB |
| CXCL5 | IL8RA |
| CXCL5 | IL8RB |
| CXCL6 | IL8RA |
| CXCL6 | IL8RB |

| | |
|---|---|
| CXCL9 | CCR3 |
| CXCL9 | CXCR3 |
| DCN | TGFB1 |
| DHH | PTCH1 |
| DKK1 | LRP5 |
| DKK1 | LRP6 |
| DKK2 | LRP6 |
| DLL4 | NOTCH1 |
| DLL4 | NOTCH4 |
| DSC2 | DSG2 |
| EDN1 | EDNRB |
| EFNA1 | EPHA1 |
| EFNA1 | EPHA2 |
| EFNA1 | EPHA3 |
| EFNA1 | EPHA4 |
| EFNA1 | EPHA7 |
| EFNA1 | EPHA8 |
| EFNA2 | EPHA2 |
| EFNA2 | EPHA3 |
| EFNA3 | EPHA2 |
| EFNA3 | EPHA4 |
| EFNA3 | EPHA7 |
| EFNA4 | EPHA2 |
| EFNA4 | EPHA4 |
| EFNA4 | EPHA7 |
| EFNA4 | EPHA8 |
| EFNA5 | EPHA2 |
| EFNA5 | EPHA3 |
| EFNA5 | EPHA4 |
| EFNA5 | EPHA7 |
| EFNA5 | EPHA8 |
| EFNB1 | EPHB1 |
| EFNB1 | EPHB2 |
| EFNB2 | EPHB1 |
| EFNB2 | EPHB2 |
| EFNB2 | EPHB4 |
| EFNB2 | EPHB6 |
| EFNB3 | EPHB3 |
| EGF | EGFR |
| EGF | ERBB2 |
| EPHA1 | EFNA1 |
| EPHA2 | EFNA1 |
| EPHA2 | EFNA2 |
| EPHA2 | EFNA3 |
| EPHA2 | EFNA4 |
| EPHA2 | EFNA5 |

| | |
|---|---|
| EPHA3 | EFNA1 |
| EPHA3 | EFNA2 |
| EPHA3 | EFNA5 |
| EPHA4 | EFNA1 |
| EPHA4 | EFNA3 |
| EPHA4 | EFNA4 |
| EPHA4 | EFNA5 |
| EPHA7 | EFNA1 |
| EPHA7 | EFNA3 |
| EPHA7 | EFNA4 |
| EPHA7 | EFNA5 |
| EPHA8 | EFNA1 |
| EPHA8 | EFNA4 |
| EPHA8 | EFNA5 |
| EPHB1 | EFNB1 |
| EPHB1 | EFNB2 |
| EPHB2 | EFNB1 |
| EPHB2 | EFNB2 |
| EPHB3 | EFNB3 |
| EPHB4 | EFNB2 |
| EPHB6 | EFNB2 |
| EPO | EPOR |
| EREG | EGFR |
| EREG | ERBB4 |
| F12 | KLKB1 |
| F2 | F11 |
| F2 | F13A1 |
| F2 | F2R |
| F2 | F2RL1 |
| F2 | F2RL2 |
| F2 | F2RL3 |
| F2 | F9 |
| F2 | FGA |
| F2 | THBD |
| F7 | F10 |
| FASLG | FAS |
| FGF10 | FGFR2 |
| FGF17 | FGFR1 |
| FGF17 | FGFR2 |
| FGF17 | FGFR3 |
| FGF17 | FGFR4 |
| FGF18 | FGFR1 |
| FGF18 | FGFR2 |
| FGF18 | FGFR3 |
| FGF18 | FGFR4 |
| FGF19 | FGFR4 |

| | |
|---|---|
| FGF2 | FGFR1 |
| FGF2 | FGFR3 |
| FGF2 | FGFR4 |
| FGF23 | FGFR2 |
| FGF23 | FGFR3 |
| FGF3 | FGFR1 |
| FGF3 | FGFR2 |
| FGF3 | FGFR3 |
| FGF3 | FGFR4 |
| FGF4 | FGFR1 |
| FGF4 | FGFR2 |
| FGF4 | FGFR3 |
| FGF4 | FGFR4 |
| FGF5 | FGFR1 |
| FGF5 | FGFR2 |
| FGF5 | FGFR3 |
| FGF5 | FGFR4 |
| FGF6 | FGFR1 |
| FGF6 | FGFR2 |
| FGF6 | FGFR3 |
| FGF6 | FGFR4 |
| FGF7 | FGFR1 |
| FGF7 | FGFR2 |
| FGF7 | FGFR3 |
| FGF7 | FGFR4 |
| FGF9 | FGFR1 |
| FGF9 | FGFR2 |
| FGF9 | FGFR3 |
| FGF9 | FGFR4 |
| FIGF | FLT4 |
| FIGF | KDR |
| FLT3LG | FLT3 |
| FN1 | ITGA3 |
| FN1 | ITGA4 |
| FN1 | ITGA5 |
| FN1 | ITGA8 |
| FN1 | ITGB1 |
| FN1 | ITGB3 |
| FN1 | ITGB6 |
| FN1 | ITGB7 |
| FN1 | SDC2 |
| FSHB | FSHR |
| FST | INHBA |
| FST | INHBE |
| GDF5 | ACVR2A |
| GDF5 | BMPR1A |

| | |
|---|---|
| GDF5 | BMPR2 |
| GDF6 | BMPR2 |
| GH2 | GHR |
| GNRH1 | GNRHR |
| HGF | MET |
| ICAM1 | ITGB2 |
| ICOSLG | ICOS |
| IFNA1 | IFNAR1 |
| IFNA2 | IFNAR1 |
| IFNA2 | IFNAR2 |
| IFNA5 | IFNAR2 |
| IFNA8 | IFNAR2 |
| IFNB1 | IFNAR1 |
| IFNB1 | IFNAR2 |
| IFNG | IFNGR1 |
| IFNG | IFNGR2 |
| IFNW1 | IFNAR1 |
| IGF1 | IGF1R |
| IHH | PTCH1 |
| IL10 | IL10RA |
| IL10 | IL10RB |
| IL11 | IL11RA |
| IL12B | IL12RB1 |
| IL12B | IL12RB2 |
| IL13 | IL13RA1 |
| IL13 | IL4R |
| IL17A | IL17RA |
| IL18 | IL18R1 |
| IL18 | IL18RAP |
| IL19 | IL20RA |
| IL1B | IL1R1 |
| IL1B | IL1R2 |
| IL1B | IL1RAP |
| IL2 | IL2RA |
| IL2 | IL2RB |
| IL2 | IL2RG |
| IL20 | IL20RA |
| IL21 | IL21R |
| IL22 | IL10RB |
| IL22 | IL22RA1 |
| IL22 | IL22RA2 |
| IL23A | IL12RB1 |
| IL24 | IL20RA |
| IL28A | IL10RB |
| IL28A | IL28RA |
| IL28B | IL10RB |

| | |
|---|---|
| IL29 | IL28RA |
| IL3 | CSF2RB |
| IL3 | IL3RA |
| IL4 | IL2RG |
| IL4 | IL4R |
| IL5 | IL5RA |
| IL6 | IL6R |
| IL6 | IL6ST |
| IL7 | IL2RG |
| IL7 | IL7R |
| IL8 | IL8RA |
| IL8 | IL8RB |
| IL9 | IL9R |
| INHBA | ACVR1 |
| INHBA | ACVR1B |
| INHBA | ACVR2A |
| INHBA | ACVR2B |
| INHBB | ACVR1 |
| INHBB | ACVR1B |
| INHBB | ACVR2A |
| INHBB | ACVR2B |
| INHBC | ACVR1 |
| INHBC | ACVR1B |
| INHBC | ACVR2A |
| INHBC | ACVR2B |
| INS | IGF1R |
| INS | INSR |
| ICAM3 | ITGAL |
| ITGAM | ICAM1 |
| ICAM2 | ITGAM |
| ITGAM | JAM3 |
| ITGB1 | VCAM1 |
| ITGB2 | CD226 |
| ITGB2 | ICAM2 |
| ITGB2 | ICAM3 |
| JAG1 | NOTCH1 |
| JAG1 | NOTCH2 |
| JAG1 | NOTCH3 |
| JAG2 | NOTCH1 |
| JAG2 | NOTCH2 |
| JAG2 | NOTCH3 |
| JAM2 | JAM3 |
| KITLG | KIT |
| KLKB1 | KNG1 |
| L1CAM | NCAM1 |
| LAMA1 | DAG1 |

| | |
|---|---|
| LAMA1 | ITGA1 |
| LAMA1 | ITGA2 |
| LAMA1 | ITGB1 |
| LAMA2 | DAG1 |
| LAMA5 | DAG1 |
| LEP | LEPR |
| LHB | LHCGR |
| LTA | LTBR |
| LTA | TNFRSF14 |
| LTA | TNFRSF1A |
| LTA | TNFRSF1B |
| LTB | LTBR |
| MADCAM1 | ITGA4 |
| MADCAM1 | ITGB7 |
| MASP1 | MBL2 |
| MBL2 | MASP2 |
| MFNG | NOTCH1 |
| MFNG | NOTCH2 |
| MMP14 | MMP2 |
| NGF | NGFR |
| NGF | NTRK1 |
| NOG | BMP2 |
| NOG | BMP4 |
| NOG | BMP5 |
| NOG | BMP7 |
| NRG1 | ERBB3 |
| NRG1 | ERBB4 |
| NRG3 | ERBB4 |
| NRXN1 | NLGN1 |
| NRXN1 | NLGN2 |
| NRXN1 | NLGN3 |
| NRXN2 | NLGN3 |
| NRXN3 | NLGN3 |
| NTF4 | NGFR |
| NTN1 | DCC |
| NTN1 | UNC5C |
| OSM | IL6ST |
| OSM | LIFR |
| OSM | OSMR |
| PDGFA | PDGFRA |
| PDGFB | PDGFRA |
| PDGFB | PDGFRB |
| PDGFC | PDGFRA |
| PDGFD | PDGFRB |
| PF4 | CXCR3 |
| PGF | FLT1 |

| | |
|---|---|
| PLAT | PLG |
| PLAU | PLAUR |
| PLG | F2R |
| PLG | F2RL1 |
| POMC | MC1R |
| PPBP | IL8RA |
| PPBP | IL8RB |
| PRL | PRLR |
| PROC | F5 |
| PROC | F8 |
| PROS1 | F5 |
| PROS1 | F8 |
| PVR | CD226 |
| PVRL2 | CD226 |
| PVRL2 | PVRL3 |
| SELPLG | SELE |
| SELPLG | SELP |
| SEMA3A | NRP1 |
| SEMA3B | NRP1 |
| SEMA3C | NRP1 |
| SEMA3F | NRP1 |
| SEMA4B | PLXNB1 |
| SEMA4D | PLXNB1 |
| SEMA7A | PLXNC1 |
| SERPINA5 | F2 |
| SERPINA5 | PROC |
| SERPINC1 | F2 |
| SERPIND1 | F2 |
| SERPINE1 | PLAT |
| SERPINE1 | PLAU |
| SERPINF2 | PLG |
| SFRP1 | FZD6 |
| SFRP1 | WNT1 |
| SFRP1 | WNT2 |
| SFRP1 | WNT4 |
| SFRP2 | WNT1 |
| SFRP2 | WNT4 |
| SHH | PTCH1 |
| SLIT1 | ROBO1 |
| SLIT2 | ROBO1 |
| SPP1 | ITGA5 |
| SPP1 | ITGA9 |
| SPP1 | ITGAV |
| SPP1 | ITGB1 |
| SST | SSTR2 |
| TFPI | F10 |

| | |
|---|---|
| TGFB1 | TGFBR1 |
| TGFB2 | TGFBR1 |
| TGFB3 | TGFBR1 |
| THBS1 | CD36 |
| THBS1 | CD47 |
| THBS1 | ITGB3 |
| THY1 | ITGAM |
| TNC | ITGA5 |
| TNF | TNFRSF1A |
| TNF | TNFRSF1B |
| TNFRSF17 | TNFSF13 |
| TNFSF10 | TNFRSF10B |
| TNFSF10 | TNFRSF10C |
| TNFSF10 | TNFRSF10D |
| TNFSF11 | TNFRSF11A |
| TNFSF12 | TNFRSF12A |
| TNFSF13 | TNFRSF13B |
| TNFSF13B | TNFRSF13B |
| TNFSF13B | TNFRSF13C |
| TNFSF13B | TNFRSF17 |
| TNFSF14 | LTBR |
| TNFSF14 | TNFRSF14 |
| TNFSF4 | TNFRSF4 |
| TNFSF8 | TNFRSF8 |
| TNFSF9 | TNFRSF9 |
| TSLP | CRLF2 |
| TSLP | IL7R |
| VCAM1 | ITGB7 |
| VEGFA | FLT1 |
| VEGFA | KDR |
| VEGFB | FLT1 |
| VEGFC | FLT4 |
| VEGFC | KDR |
| VTN | ITGA8 |
| VTN | ITGB3 |
| VTN | ITGB6 |
| VTN | ITGB8 |
| VWF | GP1BA |
| VWF | ITGA2B |
| WNT1 | FZD8 |
| WNT1 | FZD9 |
| WNT2 | FZD1 |
| WNT2 | FZD9 |
| WNT3 | FZD1 |
| WNT3A | FZD1 |
| WNT4 | FZD6 |

| | |
|---|---|
| WNT5A | FZD1 |
| WNT5A | FZD5 |
| WNT7A | FZD5 |
| WNT7A | FZD9 |
| ADAM12 | EGF |
| ANGPT1 | TEK |
| ANGPT1 | ITGA5 |
| ANGPT2 | TEK |
| ANGPT4 | TEK |
| ANGPTL1 | TEK |
| ANGPTL3 | ITGB3 |
| ANGPTL3 | ITGAV |
| ANGPTL3 | ITGA5 |
| BMP15 | BMPR1B |
| BMP2 | ENG |
| BMP2 | ACVR2B |
| BMP5 | BMPR1A |
| BMP7 | ACVR2B |
| BMP7 | ACVR1 |
| BMP7 | ENG |
| CHRDL2 | BMP4 |
| CHRDL2 | BMP5 |
| CHRDL2 | BMP2 |
| CHRDL2 | BMP7 |
| DHH | PTCH2 |
| DKK1 | KREMEN1 |
| GAL | GALR2 |
| GDF5 | BMPR1B |
| GDF6 | BMPR1A |
| GDF6 | BMPR1B |
| GDF9 | BMPR2 |
| GDF9 | ACVR2A |
| WNT3A | GPC3 |
| FGF2 | GPC4 |
| GREM2 | BMP4 |
| HGF | SDC1 |
| IGFBP2 | IGF1 |
| IHH | PTCH2 |
| IL4 | IL13RA2 |
| INHA | TGFBR3 |
| INHBA | TGFBR3 |
| ITGAV | PLP1 |
| MEP1A | NTS |
| PGF | NRP2 |
| SEMA3B | NRP2 |
| SEMA3F | NRP2 |

| | |
|---|---|
| SEMA4A | PLXND1 |
| SEMA4D | CD72 |
| SEMA4D | PTPRC |
| SEMA4F | NRP2 |
| TDGF1 | ACVR1C |
| TGFB1 | ACVRL1 |
| TGFB1 | ENG |
| TGFB1 | TGFBR3 |
| TGFB2 | ENG |
| TGFB2 | TGFBR3 |
| TGFB3 | ENG |
| TGFB3 | TGFBR3 |
| TGFB3 | ACVRL1 |
| WNT1 | LRP5 |
| WNT1 | LRP6 |
| WNT3A | LRP6 |
| WNT5A | ROR2 |
| WNT7B | GPC3 |
| ADAM12 | EGF |
| ANGPT1 | TEK |
| ANGPT1 | ITGA5 |
| ANGPT2 | TEK |
| ANGPT4 | TEK |
| ANGPTL1 | TEK |
| ANGPTL3 | ITGB3 |
| ANGPTL3 | ITGAV |
| ANGPTL3 | ITGA5 |
| BMP15 | BMPR1B |
| BMP2 | ENG |
| BMP2 | ACVR2B |
| BMP5 | BMPR1A |
| BMP7 | ACVR2B |
| BMP7 | ACVR1 |
| BMP7 | ENG |
| CHRDL2 | BMP4 |
| CHRDL2 | BMP5 |
| CHRDL2 | BMP2 |
| CHRDL2 | BMP7 |
| DHH | PTCH2 |
| DKK1 | KREMEN1 |
| GAL | GALR2 |
| GDF5 | BMPR1B |
| GDF6 | BMPR1A |
| GDF6 | BMPR1B |
| GDF9 | BMPR2 |
| GDF9 | ACVR2A |

| | |
|---|---|
| WNT3A | GPC3 |
| FGF2 | GPC4 |
| GREM2 | BMP4 |
| HGF | SDC1 |
| IGFBP2 | IGF1 |
| IHH | PTCH2 |
| IL4 | IL13RA2 |
| INHA | TGFBR3 |
| INHBA | TGFBR3 |
| ITGAV | PLP1 |
| MEP1A | NTS |
| PGF | NRP2 |
| SEMA3B | NRP2 |
| SEMA3F | NRP2 |
| SEMA4A | PLXND1 |
| SEMA4D | CD72 |
| SEMA4D | PTPRC |
| SEMA4F | NRP2 |
| TDGF1 | ACVR1C |
| TGFB1 | ACVRL1 |
| TGFB1 | ENG |
| TGFB1 | TGFBR3 |
| TGFB2 | ENG |
| TGFB2 | TGFBR3 |
| TGFB3 | ENG |
| TGFB3 | TGFBR3 |
| TGFB3 | ACVRL1 |
| WNT1 | LRP5 |
| WNT1 | LRP6 |
| WNT3A | LRP6 |
| WNT5A | ROR2 |
| WNT7B | GPC3 |
| PECAM1 | CD38 |

**system.**
