# Supplementary material for: CASTIN: a system for comprehensive analysis of cancer-stromal interactome
Source: BMC Genomics. 2016 Nov 9;17:899. doi: 10.1186/s12864-016-3207-z (PMC5103609; doi:10.1186/s12864-016-3207-z)

# CASTIN viewer

**Input File**

Choose File No file chosen

**Threshold of signal strength**

8

**Search Genes(Gene Symbol)**

View

## Cancer cell-ligand → Stromal receptor

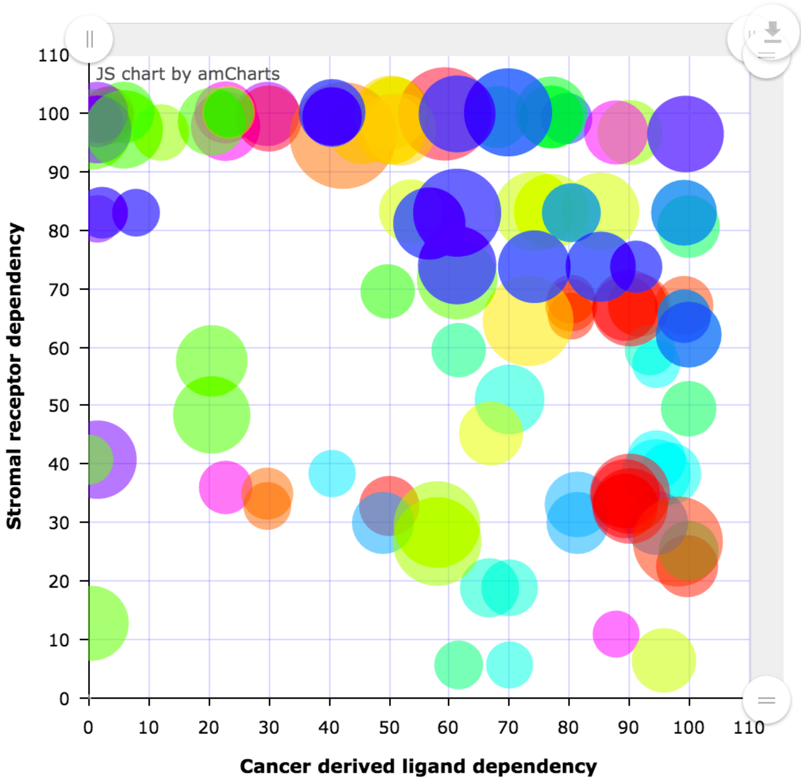


## Stromal ligand → Cancer cell-receptor

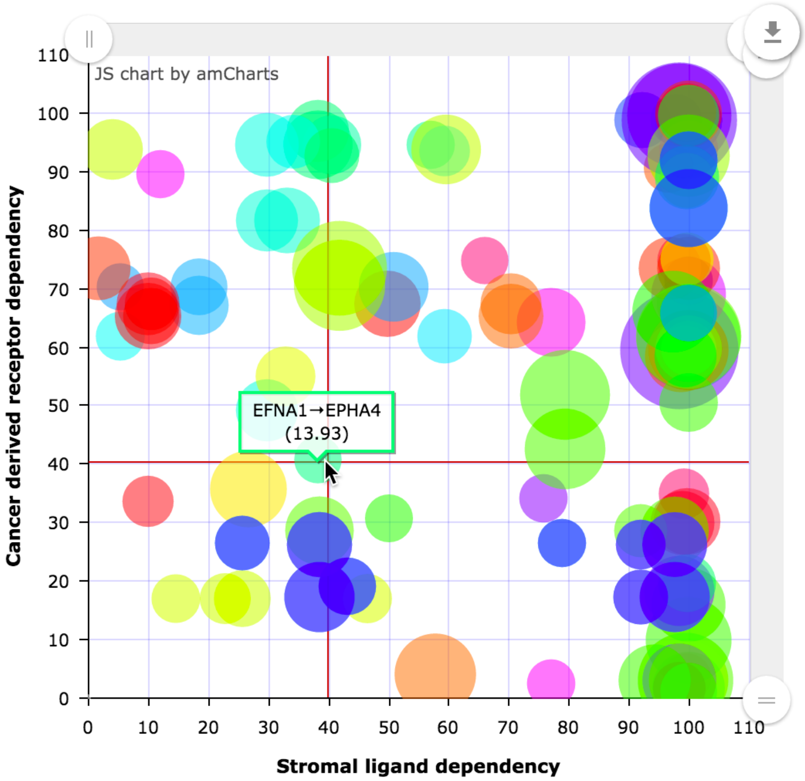

Supplement: Additional file 3: Figure S2. — Screenshot of the CASTIN interactive viewer. Hover a cursor over a circle and it displays the interacting genes represented and their value of signal strength. (PDF 1376 kb) [file 12864_2016_3207_MOESM3_ESM.pdf]
