# Supplementary material for: CASTIN: a system for comprehensive analysis of cancer-stromal interactome
Source: BMC Genomics. 2016 Nov 9;17:899. doi: 10.1186/s12864-016-3207-z (PMC5103609; doi:10.1186/s12864-016-3207-z)
Supplement: Additional file 4: Table S2. — Summary statistics of RNA-seq for mixture of total RNA from human (PANC-1) and mouse (SVEC4-10) cell lines. (PDF 37 kb) [file 12864_2016_3207_MOESM4_ESM.pdf]

Supplementary Table 2. Summary statistics of RNA-seq for mixture [illegible]

Sample	RNA ratio(mouse / human)	Total reads	Human reads
ExpID-523	75%	39940114	6503840
ExpID-524	50%	38247984	11657362
ExpID-525	25%	37906700	17062833

of total RNA from human (PANC-1) and mouse (SVEC4-10) cell lines

Mouse reads	Mouse reads (%)
16031194	71.1%
11120706	48.8%
4584808	21.2%
