# Supplementary material for: CASTIN: a system for comprehensive analysis of cancer-stromal interactome
Source: BMC Genomics. 2016 Nov 9;17:899. doi: 10.1186/s12864-016-3207-z (PMC5103609; doi:10.1186/s12864-016-3207-z)
Supplement: Additional file 8: Figure S6. — Distribution of mutually dependent interactions with strong signals in PDAC samples. a) Stromal SEMA3C to cancer-cell PLXNB1. b) Cancer-cell SEMA4D to stromal NRP1. (PDF 136 kb) [file 12864_2016_3207_MOESM8_ESM.pdf]

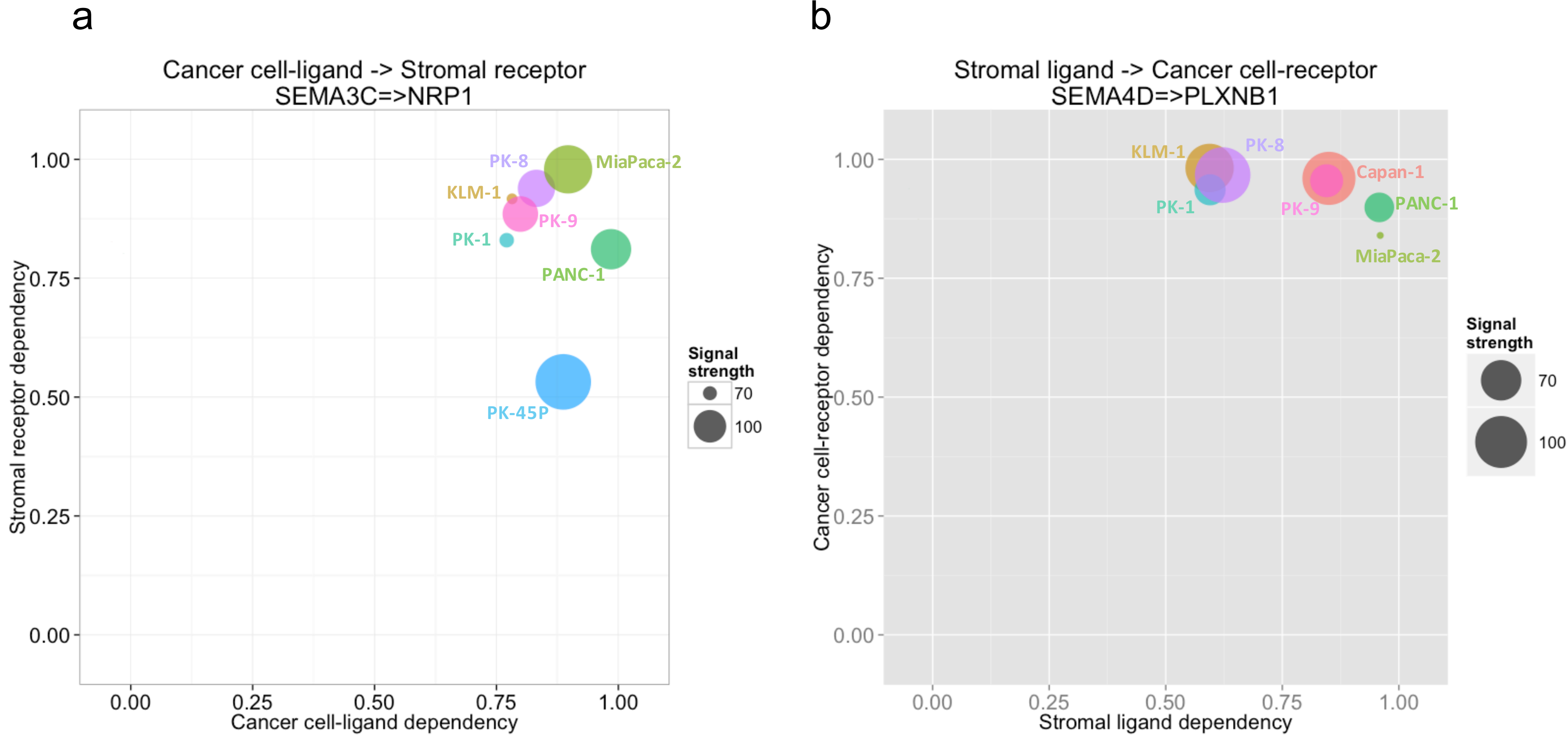
a
Cancer cell-ligand -> Stromal receptor
SEMA3C=>NRP1
Stromal receptor dependency
Cancer cell-ligand dependency
0.00
0.25
0.50
0.75
1.00
PK-8
MiaPaca-2
KLM-1
PK-9
PK-1
PANC-1
PK-45P
Signal strength
70
100
b
Stromal ligand -> Cancer cell-receptor
SEMA4D=>PLXNB1
Cancer cell-receptor dependency
Stromal ligand dependency
KLM-1
PK-8
Capan-1
PK-1
PK-9
PANC-1
MiaPaca-2
Signal strength
70
100
